# Conservation and Diversification of an Ancestral Chordate Gene Regulatory Network for Dorsoventral Patterning

**DOI:** 10.1371/journal.pone.0014650

**Published:** 2011-02-03

**Authors:** Iryna Kozmikova, Jana Smolikova, Cestmir Vlcek, Zbynek Kozmik

**Affiliations:** Institute of Molecular Genetics, Prague, Czech Republic; Katholieke Universiteit Leuven, Belgium

## Abstract

Formation of a dorsoventral axis is a key event in the early development of most animal embryos. It is well established that bone morphogenetic proteins (Bmps) and Wnts are key mediators of dorsoventral patterning in vertebrates. In the cephalochordate amphioxus, genes encoding Bmps and transcription factors downstream of Bmp signaling such as Vent are expressed in patterns reminiscent of those of their vertebrate orthologues. However, the key question is whether the conservation of expression patterns of network constituents implies conservation of functional network interactions, and if so, how an increased functional complexity can evolve. Using heterologous systems, namely by reporter gene assays in mammalian cell lines and by transgenesis in medaka fish, we have compared the gene regulatory network implicated in dorsoventral patterning of the basal chordate amphioxus and vertebrates. We found that Bmp but not canonical Wnt signaling regulates promoters of genes encoding homeodomain proteins AmphiVent1 and AmphiVent2. Furthermore, *AmphiVent1* and *AmphiVent2* promoters appear to be correctly regulated in the context of a vertebrate embryo. Finally, we show that AmphiVent1 is able to directly repress promoters of *AmphiGoosecoid* and *AmphiChordin* genes. Repression of genes encoding dorsal-specific signaling molecule Chordin and transcription factor Goosecoid by *Xenopus* and zebrafish Vent genes represents a key regulatory interaction during vertebrate axis formation. Our data indicate high evolutionary conservation of a core Bmp-triggered gene regulatory network for dorsoventral patterning in chordates and suggest that co-option of the canonical Wnt signaling pathway for dorsoventral patterning in vertebrates represents one of the innovations through which an increased morphological complexity of vertebrate embryo is achieved.

## Introduction

Establishment of a dorsoventral (DV) axis is a key event in early development of any bilaterian animal embryo. The crucial step in DV axis formation is specification of the dorsal and ventral mesoderm. In vertebrates, the establishment of the organizer involves activation of several genes [1]. Their protein products are mostly transcription factors (such as Otx2, XFD1, Goosecoid) or secreted proteins (such as ADMP, Nodal, Noggin, Chordin). The organizer secreted proteins Chordin and Noggin are capable to inactivate BMP signaling molecules that play a key role in the induction and maintainance of ventral and lateral mesoderm. Conversely, the expression of Chordin is negatively regulated by Bmp2 and Bmp4 proteins through their targets, ventralizing homeobox genes *Vent* and *Vox* [1]. Recently it was demonstrated that the expression patterns of genes mediating DV patterning in early development are highly conserved between basal chordates (cephalochordate amphioxus) and vertebrates [2]. Orthologues of the vertebrate organizer-specific genes such as *Goosecoid, Chordin, Nodal* are expressed in early chordate embryo [2]. Amphioxus ventral-specific genes encoding Bmp signaling molecules, and Hex, Evx and Vent transcription factors demonstrate expression patterns homologous to their vertebrate counterparts [2], [3]. It was shown previously that teleost and amphibian Vent proteins can suppress the expression of dorsal genes during early development [4], [5], [6]. Xvent-2 (also known as Xvent-2B, Xom, Xbr-1 and Vox) directly represses the *Goosecoid* promoter in *Xenopus* embryo [6]. Noting mutually exclusive expression of *AmphiVent1* and *AmphiChordin* [2], it can be suggested that AmphiVent1 is likewise able to antagonize expression of organizer-specific genes as do its vertebrate homologues [7]. During the gastrula stage, *AmphiVent1* is expressed throughout the mesendoderm [3]. By late gastrula, it is down-regulated ventrally but remains expressed dorsolaterally in the paraxial mesoderm. Then, at the mid-neurula stage, AmphiVent1-expressing ventral mesoderm forms as outgrowth from the somites [3], [8]. The developmental expression of amphibian and teleost Vent genes during gastrula stages is most conspicuous in ventral mesoderm and is down-regulated in the regions of organizer, chordamesoderm and neural plate [5], [7]. At the neurula stage, amphioxus as well as vertebrate Vent genes are expressed along the edges of the neural plate, in the tail bud/proctodeal region, and in the foregut [3], [9]. Even though there appears to be a temporal difference between the ventral expression of *AmphiVent1* and vertebrate Vent genes during early development, their dorsal expression is similar as exemplified by downregulation at the dorsal lip of the blastopore and neural plate [3]. It is interesting to note that within the animal kingdom Vent genes are present in chordates only. Moreover, although in humans the Vent-like homeobox gene has been described, no Vent gene has been found in the mouse. Amphioxus genome contains two Vent genes, which are situated on the same chromosome in close proximity of each other [10].

Multiple transcriptional inputs are likely required for the correct regulation of Vent genes. Among those Bmp-mediated activation of vertebrate Vent genes is well documented. Bmp2 and Bmp4 activate *Xvent-2* promoter via Smad1 in *Xenopus* [11], [12] and in P19 murine embryonal cells [13]. This activation is mediated synergistically by OAZ zinc finger transcription factor, which can interact with MH2 domains of Smad1 and Smad4 proteins in response to the Bmp signal [13]. Recent investigations reveal that in addition to Bmp, the canonical Wnt signaling pathway plays an important role in patterning of ventral mesoderm in *Xenopus* and zebrafish. In the zebrafish embryo Wnt8 directly activates *Vent* and *Vox* genes through β-catenin [14]. Both *Xenopus Xvent-1* and *Xvent-2* genes contain conserved Lef/Tcf binding sites in the promoter. Xwnt-8 protein can activate *Xvent-1* promoter and the activation depends on the functional Lef/Tcf binding site [15]. Likewise, transgenic analysis of *Xvent-2* promoter revealed that mutation of the Lef/Tcf binding site decreases expression of the reporter gene [16].

In this study we have investigated the role of AmphiVent1 homeodomain protein in the molecular events responsible for DV patterning in amphioxus. We have specifically focused on three main areas: the role of Bmp and canonical Wnt signaling in *AmphiVent1* gene regulation, functional properties of Vent proteins, and identification of direct targets of AmphiVent1 transcription factor. Using luciferase reporter assays in P19 murine embryonal cells we have demonstrated Bmp-mediated activation of the *AmphiVent1* 5′genomic non-coding regions (putative promoter) via Smad1/Smad4 proteins. Similar to vertebrate *Xvent-2B* gene promoter, *AmphiVent1* promoter responsiveness to Bmp signaling is co-stimulated by zinc finger transcription factor OAZ. Furthermore, reporter plasmids where expression of GFP is controlled by Xenopus *Xvent-2B* and amphioxus *AmphiVent1* promoters show a highly similar expression pattern in transgenic medaka embryos. We found that AmphiVent1 protein acts as a transcriptional repressor with the repression domain located at its N-terminus that appears to interact with groucho family co-repressor Grg4. As in the case of its vertebrate orthologues, AmphiVent1 protein can suppress the activity of amphioxus *Chordin* and *Goosecoid* gene promoters. Our data thus provide evidence for a remarkable conservation of Bmp-triggered gene regulatory network mediating DV patterning in vertebrates and basal chordates. On the other hand, our data suggest an increased complexity of DV pattern regulation in vertebrates. The canonical Wnt signaling regulatory input for ventral-specific gene expression appears to be lacking in cephalochordates (this study) [17] and has likely been co-opted in vertebrates.

## Results

### 5′genomic non-coding regions of amphioxus Vent genes are activated by Bmp signaling

**Figure 1:**
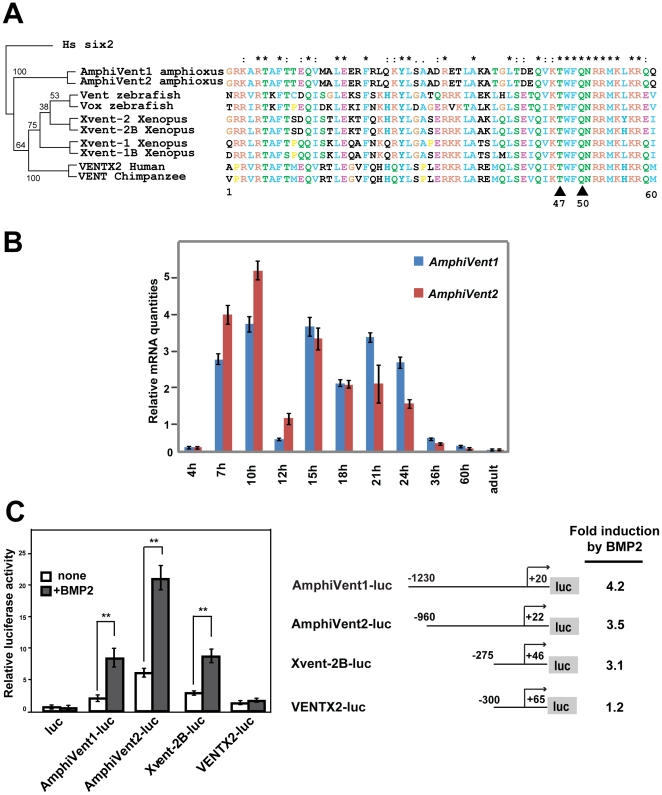
Amphioxus Vent genes are regulated by BMP signaling. (A) Phylogenetic analysis of Vent genes in the chordate lineage. Please, note that species-specific duplication is responsible for the increased copy number of Vent genes. Amino acid sequence alignment of Vent homeodomains is shown with characteristic amino acids Q50 and T47 marked by arrowheads. Numbers shown indicate bootstrap support values. (B) Quantitative RT-PCR expression analysis of *AmphiVent1* and *AmphiVent2* during *B. floridae* development. (C) 5′genomic non-coding regions of Amphioxus and *Xenopus* Vent genes are regulated by Bmp signaling. P19 cells were transfected with luciferase reporters containing *AmphiVent1*, *AmphiVent2*, *Xvent-2B* and *VENTX2* 5′genomic non-coding regions in the absence (open bars) and presence (black bars) of exogenous human BMP2. **P<0.01.

Two Vent-like genes, *AmphiVent1* [3] and *AmphiVent2* [10], can be identified in the genome of cephalochordate amphioxus (http://genome.jgi-psf.org/Brafl1/Brafl1.home.html). Both of them encode the Q50 homeodomain protein with the Vent-specific T47 substitution [18] (Fig. 1A). Likewise, two Vent genes are present in the zebrafish genome while four Vent genes are present in *Xenopus laevi*s. Higher number of Vent genes in *Xenopus laevis* may be caused by a recent duplication of its genome as only two Vent genes are found in another frog *Xenopus tropicalis* (http://www.ensembl.org). Only a single Vent gene is found in the genome of humans and chimpanzee. Interestingly, a functional copy of a Vent gene has been lost from the mouse genome; only a fragment of the Vent-type homeodomain in the mouse genome can be identified (this study; see discussion). Phylogenetic analysis suggests that independent lineage-specific duplication is responsible for the increased copy number of Vent genes (Fig. 1A). Recent lineage-specific duplication of *AmphiVent1* and *AmphiVent2* is consistent with high sequence similarity (83% nucleotide identity within respective ORF's; EMBOSS Pairwise Alignment at http://www.ebi.ac.uk/Tools/emboss/align/). Since *AmphiVent2* has not been previously characterized at all we performed an expression analysis using real-time quantitative RT-PCR. As shown in Fig. 1B *AmphiVent2* displays similar but not identical temporal and quantitative regulation of mRNA expression as compared to *AmphiVent1.*

It is well established that a relatively short (approximately 300 bp) promoter (5′genomic non-coding region) of *XVent-2B* gene is sufficient for Bmp-mediated regulation [12], [13]. Given the known position of vertebrate Vent genes in the gene regulatory network governing DV patterning, we hypothesized that amphioxus Vent genes might be regulated by Bmp signaling. To test this possibility, we isolated approximately 1 kb of 5′genomic non-coding regions of *AmphiVent1* (−1230/+20) and *AmphiVent2* (−912/+22) genes putatively containing promoters and generated luciferase reporter gene constructs. We next tested their activity in P19 cells in the absence and presence of exogenous Bmp (heterologous human BMP2 was used in this study unless indicated otherwise). The promoter of *Xenopus Xvent-2B* gene known to be activated by Bmp signaling in the embryo and in P19 cells [12], [13] was used as a control in all experiments. We observed BMP2-induced stimulation of AmphiVent1-luc and AmphiVent2-luc reporter gene activity in P19 cells that was comparable to that of Xvent-2B-luc (Fig. 1B). In contrast, the 5′genomic non-coding region (−300/+65) of the orthologous human *VENTX2* gene was not inducible by BMP2. However, it is very likely that the 5′genomic non-coding region of *VENTX2* used in our study did not contain a complete promoter and so Bmp-responsive elements might have been missing. Applying different doses of BMP2 (from 12 ng/ml up to 400 ng/ml) resulted in rather similar promoter inductions (Fig. S1A). Similar results were obtained by using human BMP4 or BMP7 for the pathway stimulation (Fig. S1B). In the same experimental setting, *AmphiVent1* promoter was not stimulated by treatment with either human TGF-β or human activin (Fig. S1C) that are, together with Bmp, members of the TGF-β super-family [19]. Combined, our data show that 5′genomic non-coding regions of *AmphiVent1* and *AmphiVent2* genes contain functional regulatory regions that are stimulated by Bmp signaling in P19 cells like their *Xenopus* counterparts and are therefore referred to as promoters in this manuscript.

### Bmp responsiveness of AmphiVent1 promoter is mediated by Smad transcription factors

**Figure 2:**
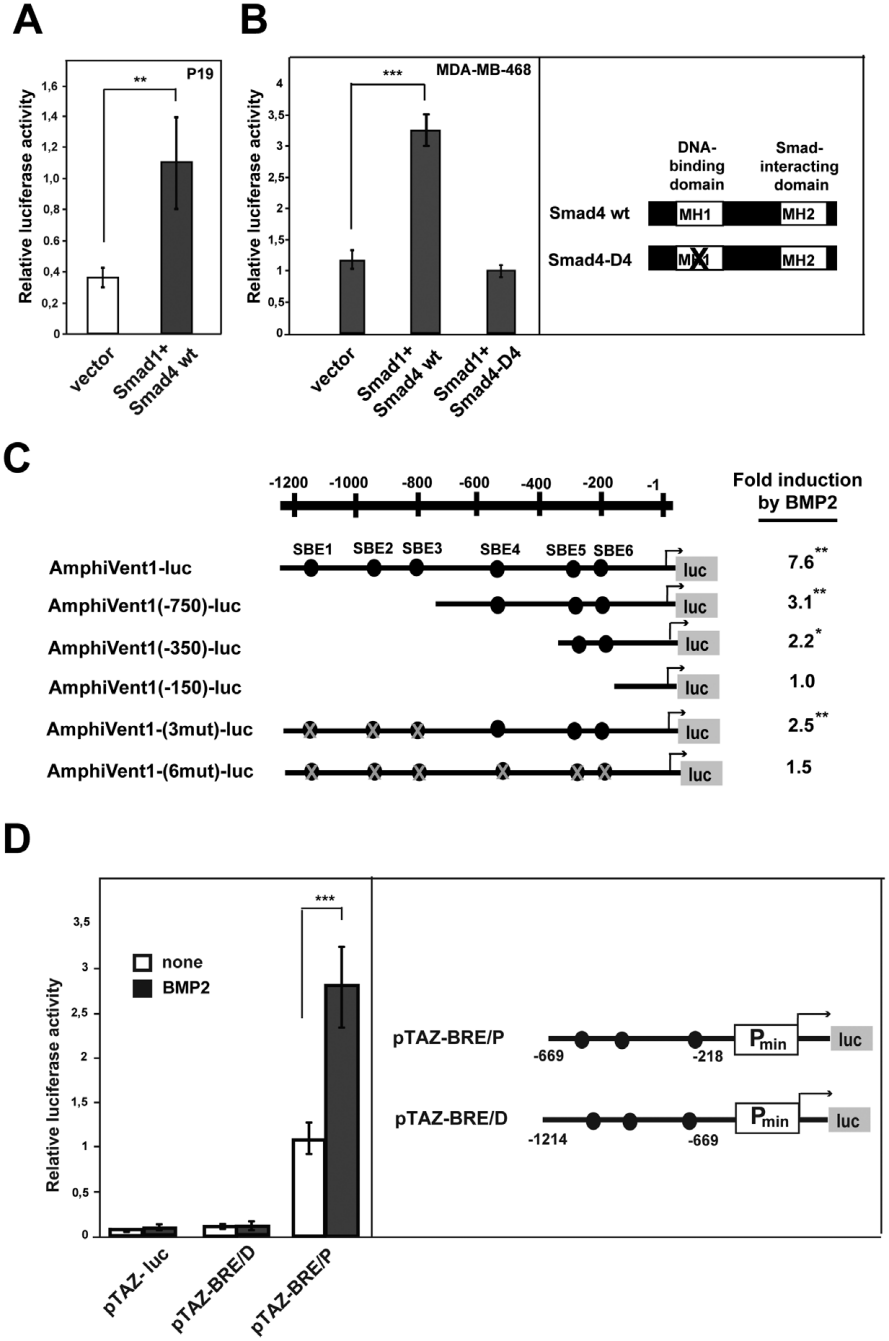
Bmp responsiveness of AmphiVent1 promoter is mediated by Smad transcription factors. (A) *AmphiVent1* reporter gene was cotransfected with or without plasmids coding Smad1 and Smad4 proteins into P19 cells. (B) *AmphiVent1* reporter gene was cotransfected with or without plasmids expressing Smad1 plus wild-type (Smad4wt) or DNA-binding-deficient mutant Smad4 (Smad4-D4) into Smad4-deficient cell line, MDA-MB-468. (C) Mapping of functional Smad-binding elements (SBE) in *AmphiVent1* promoter. Luciferase reporter plasmids containing wild-type, deleted or mutated *AmphiVent1* promoter fragments were transfected into P19 cells and cells were stimulated by BMP2. Fold-induction by BMP2 is indicated. Position of individual SBE's is indicated by black ovals, and mutated SBE's by crossed oval. (D) Identification of an autonomous BRE in *AmphiVent1* promoter. P19 cells were transfected with reporters containing a minimal promoter fused to either three proximal SBE elements (pTAZ-BRE/P) or three distal SBE elements (pTAZ-BRE/D). Reporter genes were stimulated by exogenous human BMP2 (50 ng/ml). *P<0.05, **P<0.01, ***P<0.001.

We next decided to molecularly dissect *AmphiVent1* promoter regulation. We have chosen *AmphiVent1* since the corresponding gene has previously been characterized and represents an important marker of ventral mesoderm in amphioxus [2], [3]. We tested if Bmp responsiveness of the *AmphiVent1* gene promoter is mediated by the members of the Smad group of proteins. Co-transfection of common partner human Smad4 with receptor-activated human Smad1 into P19 cells resulted in activation of the *AmphiVent1* promoter (Fig. 2A). To explore whether intrinsic Smad4 DNA-binding activity is required for promoter activation, we used a Smad4-deficient cell line, MDA-MB-468. Wild-type Smad4, but not DNA-binding-deficient mutant Smad4-D4 [20], was able to induce promoter activity when cotransfected with Smad1 (Fig. 2B). We identified six putative Smad-binding elements (SBE; cAGAC) in the promoter of *AmphiVent1* (Fig. 2C). To functionally analyze SBE's within the *AmphiVent1* promoter, 5′-truncated promoter fragments (−750/+20, −350/+20 and −150/+20) were cloned upstream of the luciferase reporter gene (Fig. 2C). These truncated reporter genes were transfected into P19 cells and cells were stimulated by BMP2. We have observed a gradual decrease of Bmp responsiveness of reporter genes that was directly correlated with the extent of promoter truncation (Fig. 2C). The stimulatory effect of BMP2 was completely abolished only in the case of AmphiVent(−150)-luc reporter gene construct, which does not contain any putative SBE's (Fig. 2C). These results show that *in vitro* all putative SBE's are relevant for Bmp-mediated inducibility of the *AmphiVent1* promoter. We next confirmed these data by a mutational analysis of the *AmphiVent1* promoter. Reporter gene constructs containing point mutations of individual SBE's within *AmphiVent1* promoter were generated. We found that destroying any single SBE does not have a significant effect on Bmp responsiveness (data not shown). Only when all six SBE's were mutated, the Bmp responsiveness of *AmphiVent1* reporter gene was completely lost (Fig. 2C). We were further interested in whether the upstream promoter region of *AmphiVent1* is able to function as an autonomous Bmp response element (BRE). Such BRE activity was previously ascribed to a specific region of *Xenopus Xvent-2B* promoter [13]. To this end two reporter gene constructs were generated that contained either a cluster of three proximal (−669/−218) or three distal (−1214/−669) SBE's upstream of a minimal promoter (constructs designated pTAZ-BRE/P and pTAZ-BRE/D, respectively). Constructs were tested for their activity in P19 cells in the absence and presence of exogenous human BMP2. Although deletion and mutation analyses revealed that all SBE's are functional within the context of the natural *AmphiVent1* promoter, only the cluster of proximal SBE's can function as an autonomous BRE when fused to a heterologous promoter (Fig. 2D). Taken together, our data suggest that the promoter of amphioxus *AmphiVent1* gene is directly activated by the Smad-mediated Bmp signaling pathway.

### Smad proteins co-operate with zinc finger protein OAZ in amphiVent1 promoter activation

**Figure 3:**
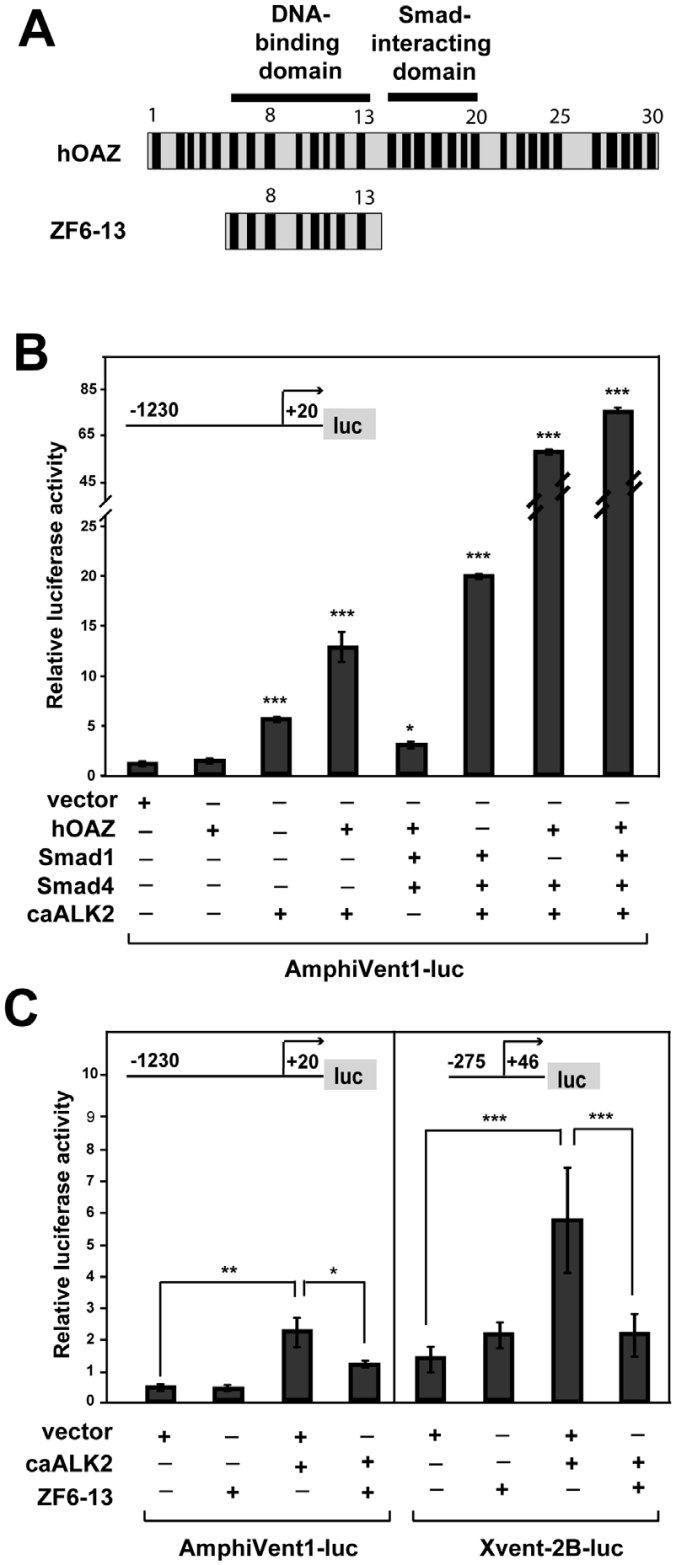
Zinc finger protein OAZ mediates induction of *AmphiVent1* promoter by BMP signaling. (A) Schematic structure of OAZ and dominant-negative construct ZF6-13. Individual zinc fingers are shown as black boxes. DNA-binding and Smad-interacting domains of OAZ are indicated. (B) OAZ potentiates Bmp-mediated induction of *AmphiVent1* promoter. P19 cells were transiently cotransfected with *AmphiVent1* reporter (−1230+20-luc) and indicated expression plasmids. (C) The dominant-negative form of OAZ attenuates Bmp inducibility of *AmphiVent1* and *Xvent-2B* promoters. *AmphiVent1* and *Xvent-2B* reporter genes were cotransfected in P19 cells with or without dominant-negative ZF6-13 construct in the absence or presence of Bmp pathway stimulation mediated by expression plasmid encoding caAlk2. *P<0.05, **P<0.01, ***P<0.001.

OAZ is a 30-zinc finger (ZF) protein, which associates with Smad1 in response to BMP2, allowing selective recognition of the BRE in *Xenopus Xvent-2* promoter [13]. ZF's 6-13 of OAZ bind directly to the BRE of *Xvent-2* promoter whereas ZF's14-19 at the C-terminus of OAZ interact with Smad1 and Smad4 (Fig. 3A). The human OAZ protein (hOAZ) is homologous to *Xenopus* and amphioxus OAZ transcription factors [13] (data not shown). To investigate whether OAZ is involved in Bmp-dependent regulation of *AmphiVent1* promoter, we cotransfected human hOAZ with constitutively active human receptor caAlk2 into P19 cells. Expression of caAlk2 is known to trigger Bmp signaling, thus mimicking addition of a Bmp ligand [21]. Transfection of hOAZ cDNA alone did not stimulate the *AmphiVent1* reporter gene. As expected, expression of the constitutively active caAlk2 alone activated the *AmphiVent1* reporter gene about 4-fold (Fig. 3B). Cotransfection of the hOAZ expression vector together with caAlk2 resulted in potentiation of caAlk2-mediated response (12-fold) (Fig. 3B), suggesting that, although OAZ is present in P19 cells [13], it appears to be a limiting factor for Bmp-dependent regulation. Finally, cotransfection of expression vectors encoding caAlk2, hOAZ, Smad1 and Smad4 into P19 cells led to remarkable activation of the *AmphiVent1* reporter gene (70- fold activation) (Fig. 3B). These results suggest that transcription factor OAZ mediates Bmp regulation of the *AmphiVent1* gene in a similar way as it does in the case of the *Xvent-2* gene. To provide an insight into the molecular mechanism of OAZ-mediated regulation of *AmphiVent1* promoter we used a dominant-negative hOAZ construct. It is known that ZF's 6-13 constitute the DNA-binding domain of hOAZ, which is however lacking the ability to interact with SMAD's and thereby to activate target promoters [13]. Based on these properties hOAZzf6-13 was previously used as a dominant-negative protein [13]. We cotransfected hOAZzf6-13 together with caAlk2 into P19 cells and we examined the responses of *Xvent-2* and *AmphiVent1* reporter genes. In congruence with our previous results, caAlk2-induced activation of *AmphiVent1* and *Xvent-2* reporter genes was significantly suppressed by cotransfection of the hOAZzf6-13 construct (Fig. 3C). The most likely explanation of the observed suppressive effect of hOAZzf6-13 is that the dominant-negative form of hOAZ competes with endogenous OAZ expressed in P19 cells for DNA-binding on *AmphiVent1* promoter. It was shown previously that the same mechanism, namely DNA-binding displacement of endogenous OAZ by hOAZzf6-13, was responsible for attenuation of Bmp-mediated activation of *Xvent-2* [13]. Summarized, our data indicate deep homology in the molecular mechanisms of Bmp-mediated regulation of chordate vent genes.

### Amphioxus Vent genes are not regulated by Wnt/β-catenin signaling

**Figure 4:**
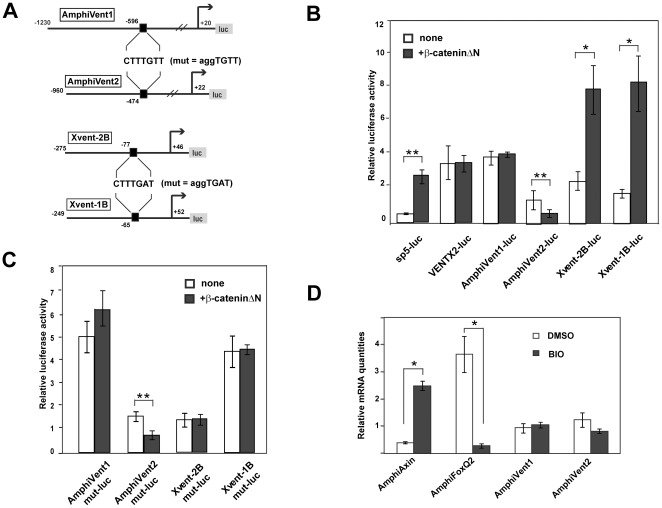
Canonical Wnt signaling activates Xenopus *Xvent-1B* and *Xvent-2B* but not *AmphiVent1* and *AmphiVent2* promoters. (A) Schematic diagram of the *Xvent-1B*, *Xvent-2B*, *AmphiVent1* and *AmphiVent2* promoter-luciferase constructs with putative Tcf/Lef binding sites depicted by black rectangles. Nucleotide changes within Tcf/Lef binding site introduced into mutant luciferase reporter genes used in (C) are indicated. (B, C) Wild-type (B) or mutant (C) luciferase reporter plasmids were cotransfected with expression plasmid encoding a stabilized form of β-catenin (β-cateninΔN) into 293T cells. Please, note that fold induction of individual reporter genes was normalized to activation of the promoter-less construct pGL3-basic. (D) Quantitative RT-PCR expression analysis of *AmphiAxin*, *AmphiFoxQ2*, *AmphiVent1* and *AmphiVent2* in control embryos (DMSO) and in embryos treated with canonical Wnt signaling activator (BIO) [23]. *P<0.05, **P<0.01, ***P<0.001.

It was shown that Wnt/β-catenin signaling directly regulates *Xvent-1B* and *Xvent-2B* genes via binding of the Lef/Tcf/β-catenin complex to their promoters [15], [16]. To analyze a plausible role in the regulation of *AmphiVent1* and *AmphiVent2* genes, we first searched for Lef/Tcf binding motifs within their promoters. We found putative Lef/Tcf elements (5′-CTTTGTT-3′) in both *AmphiVent1* (position −596/−590) and *AmphiVent2* (position −450/−446) promoters (Fig. 4A). Promoters of Xenopus Vent genes, however, contain conserved consensus Lef/Tcf binding sequences in a more proximal position (−65/−59 in *Xvent-1B* promoter and −76/−70 in *Xvent-2B* promoter, respectively) (Fig. 4A). In contrast, the 5′genomic non-coding region of human *VENTX2* (−248/+65) does not contain any Lef/Tcf binding sequence (data not shown). To examine whether promoters of *AmphiVent1*, *AmphiVent2*, *Xvent-1B* and *Xvent-2B* genes and 5′genomic non-coding region of *VENTX2* gene are responsive to canonical Wnt signaling, we cotransfected their reporter genes into 293T cells together with N-terminally truncated β-catenin (β-cateninΔN). It is well established that β-cateninΔN is a constitutively active form of β-catenin (proteolytically stabilized), which is able to interact with endogenous LEF/TCF transcription factors, thus mimicking activation of canonical (Wnt/β-catenin) signaling. Mouse *Sp5* promoter is known to be responsive to canonical Wnt signaling and was used as a positive control [22]. Only the activity of *Xvent-1B* and *Xvent-2B* promoters was significantly stimulated by cotransfection of human β-cateninΔN (Fig. 4B). Conversely, cotransfection of β-cateninΔN with AmphiVent1-luc, AmphiVent2-luc and VENTX2-luc did not lead to any significant stimulation of the respective reporter genes. On the contrary, cotransfection of β-cateninΔN with AmphiVent2-luc resulted in a modest but significant repression of the reporter gene. Similar data were obtained when Wnt3A-conditioned medium was applied to 293T cells transfected with the individual vent reporter genes (Fig. S2). Next we mutated Lef/Tcf binding sites in *AmphiVent1*, *AmphiVent2*, *Xvent-1B* and *Xvent-2B* promoters and performed cotransfections with β-cateninΔN into 293T cells. As shown in Fig. 4C, responsivness of mutated *Xvent-1B* and *Xvent-2B* promoters to β-cateninΔN was abolished indicating that single Lef/Tcf binding sites in these promoters mediate canonical Wnt signaling. As expected, mutating putative Lef/Tcf binding sites in *AmphiVent1* and *AmphiVent2* promoters did not have any significant effect in reporter gene assays as compared to wild-type constructs. Cotransfection of β-cateninΔN with AmphiVent2mut-luc (like AmphiVent2-luc, see above) resulted in a modest repression of the reporter gene suggesting an indirect type of regulation. These results confirm previously published data showing direct regulation of the two *Xenopus* vent genes by canonical Wnt signaling [15], [16]. Our data suggested that *AmphiVent1* and *AmphiVent2* genes are not directly regulated by canonical Wnt signaling. However, our conclusions were based on rather limited 5′genomic non-coding regions that might be sufficient for Bmp-responsivness but not necessarily for responsiveness to canonical Wnt signaling. To provide more definitive answer about the possible role of Wnt/β-catenin signaling in the regulation of *AmphiVent1* and *AmphiVent2* expression we pharmacologically manipulated Wnt pathway *in vivo* in the developing amphioxus embryos. To activate the canonical Wnt signaling, we used 6-Bromoindirubin-3′-oxime (BIO), a potent and less toxic inhibitor of glycogen synthase kinase-3β (GSK-3β) as compared to lithium (Li^+^)[23]. BIO was added to developing amphioxus embryos at blastula stage and embryos were allowed to develop until mid-neurula stage at which point mRNA was isolated and gene expression interrogated by real-time quantitative RT-PCR. Amphioxus FoxQ2 and Axin genes were used as controls to test for effectiveness of Wnt pathway stimulation. It was previously shown that AmphiFoxQ2 expression is downregulated upon pharmacological manipulation of canonical Wnt signaling (Li^+^ administration; [24]). Axin is a functional component of Wnt/β-catenin signaling that associates directly with β-catenin, GSK-3β and APC and is implicated in down- regulating Wnt signaling [25]. Vertebrate *Axin2* is a direct target of Wnt/β-catenin signaling whose expression is induced by activated Wnt signaling and acts therefore in a negative feedback loop [26], [27]. *Axin2* is currently the most reliable and frequently used natural readout of Wnt/β-catenin signaling *in vivo* (http://www.stanford.edu/group/nusselab/cgi-bin/wnt/reporters). As shown in Fig. 4D, constitutive activation of Wnt/β-catenin signaling in the developing amphioxus embryos resulted in marked increase of *AmphiAxin* expression and downregulation of *AmphiFoxQ2* expression. In contrast, however, expression of *AmphiVent1* and *AmphiVent2* has not been significantly upregulated in the presence of activated Wnt/β-catenin signaling.

Available evidence thus suggests that although canonical Wnt signaling plays a prominent role in the early establishment of ventral mesoderm in *Xenopus* and zebrafish, amphioxus does not use this pathway for specification of the ventral fate. In summary, our data argue that, in contrast to the situation with Bmp signaling, participation of the Wnt/β-catenin signaling pathway in regulation of Vent genes is not conserved among chordates.

### Functional diversification of amphioxus Vent gene promoters: a case of possible regulation by dorsal-specific forkhead transcription factors

**Figure 5:**
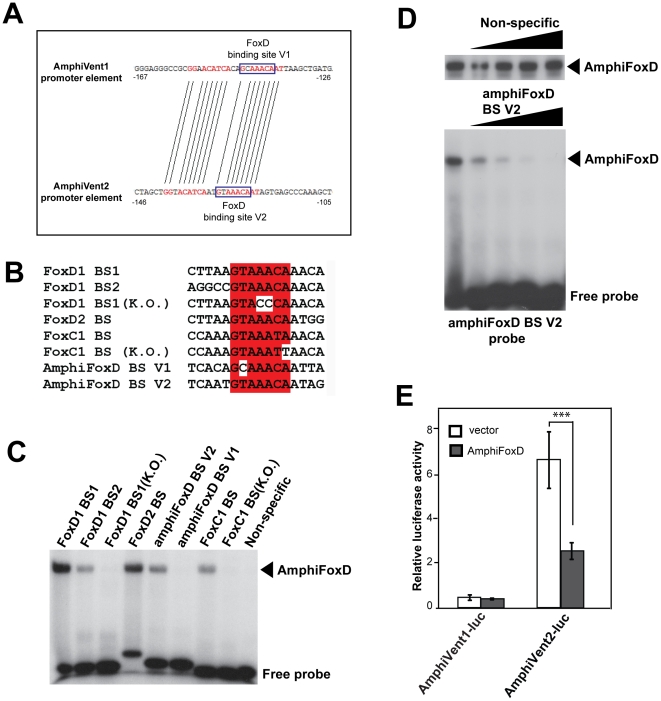
Promoter of *AmphiVent2* contains a binding site for dorsal-specific forkhead transcription factor AmphiFoxD. (A) Family Relations software was used for identification of highly conserved sequences including a putative FoxD binding site in *AmphiVent1* and *AmphiVent2* promoters. (B) Sequences of wild-type and mutated forkhead binding sites. Previously characterized binding sites for vertebrate FoxD and FoxC family members were aligned with putative sites derived from AmphiVent1 and AmphiVent2 promoters. (C) EMSA of AmphiFoxD interaction with binding sites indicated in (B). Please, note that only the binding site derived from *AmphiVent2* promoter (designated AmphiFoxD BS V2) is recognized by AmphiFoxD transcription factor. Non-specific (unrelated) double-stranded oligonucleotide is not able to bind AmphiFoxD. (D) EMSA of AmphiFoxD with binding site derived from *AmphiVent2* promoter in the presence of increasing amounts (10×, 20×, 40×, 80×) of non-specific (unrelated) double-stranded oligonucleotide or AmphiFoxD binding site. Please, note that only AmphiFoxD binding site can effectively compete for the formation of the complex. (E) P19 cells were transfected with luciferase reporters containing *AmphiVent1* or *AmphiVent2* in the presence or absence of an expression plasmid encoding AmphiFoxD. AmphiFoxD can significantly repress AmphiVent2 but not AmphiVent1 promoter. ***P<0.001.

By analyzing the proximal regions of *AmphiVent1* and *AmphiVent2* promoters *in silico* using Family Relations software (http://family.caltech.edu) we identified highly conserved sequence motifs that are 80% similar within a sliding 20 bp window (Fig. 5A and data not shown). A FoxD-binding element GTAAC was found within this region in the *AmphiVent2* gene whereas the promoter of *AmphiVent1* contains a single nucleotide change (GcAAC) in the FoxD motif (Fig. 5A). In amphioxus *AmphiFoxD* is expressed in the axial mesendoderm within the dorsal lip of blastopore at early gastrula stage [28]. Taking into account the expression pattern data and our *in silico* analysis we hypothesized that AmphiVent genes might be targets of AmphiFoxD. From this point of view, it is interesting to note that a negative regulation between FoxD and vent genes has been described in *Xenopus* [29]. *XFD-1*′, the *Xenopus* FoxD ortologue, was shown to be suppressed by the *Xvent-1* gene and plays a role in DV patterning. In fact, XFD-1′ is a dorsal lip-specific transcription factor, which is specifically activated in *Xenopus* organizer. To investigate whether AmphiFoxD protein can bind to putative sites within the conserved region of *AmphiVent1* and *AmphiVent2* promoters, double-stranded oligonucleotides derived from the corresponding regions of each promoter were tested by *in vitro* DNA-binding assay (electrophoretic mobility shift assay, EMSA). AmphiFoxD formed a specific complex with the probe, which corresponded to the *AmphiVent2* promoter region (Fig. 5B, C). In contrast, AmphiFoxD did not bind to the probe which corresponded to the *AmphiVent1* promoter region, consistent with the observed mutation in the FoxD binding motif or to a non-specific (unrelated) probe. Binding site specificity was confirmed by EMSA in the presence of increasing amounts of non-specific (unrelated) double-stranded oligonucleotide or AmphiFoxD binding site derived from *AmphiVent2* promoter region. As shown in Fig. 5D, only AmphiFoxD binding site can effectively compete for the formation of the complex. To provide further evidence for AmphiFoxD-mediated regulation of *AmphiVent2*, P19 cells were transfected with luciferase reporters containing *AmphiVent1* or *AmphiVent2* promoters in the presence or absence of an expression plasmid encoding AmphiFoxD. AmphiFoxD can significantly repress AmphiVent2-luc but not AmphiVent1-luc promoter construct (Fig. 5E). Our data suggest that *AmphiVent2,* but not *AmphiVent1*, might be subject to FoxD regulation. In addition, the data exemplify functional diversification of promoter sequences after duplication of vent genes in the amphioxus lineage.

### Activation of AmphiVent1 and AmphiVent2 promoters in early developing medaka embryos

**Figure 6:**
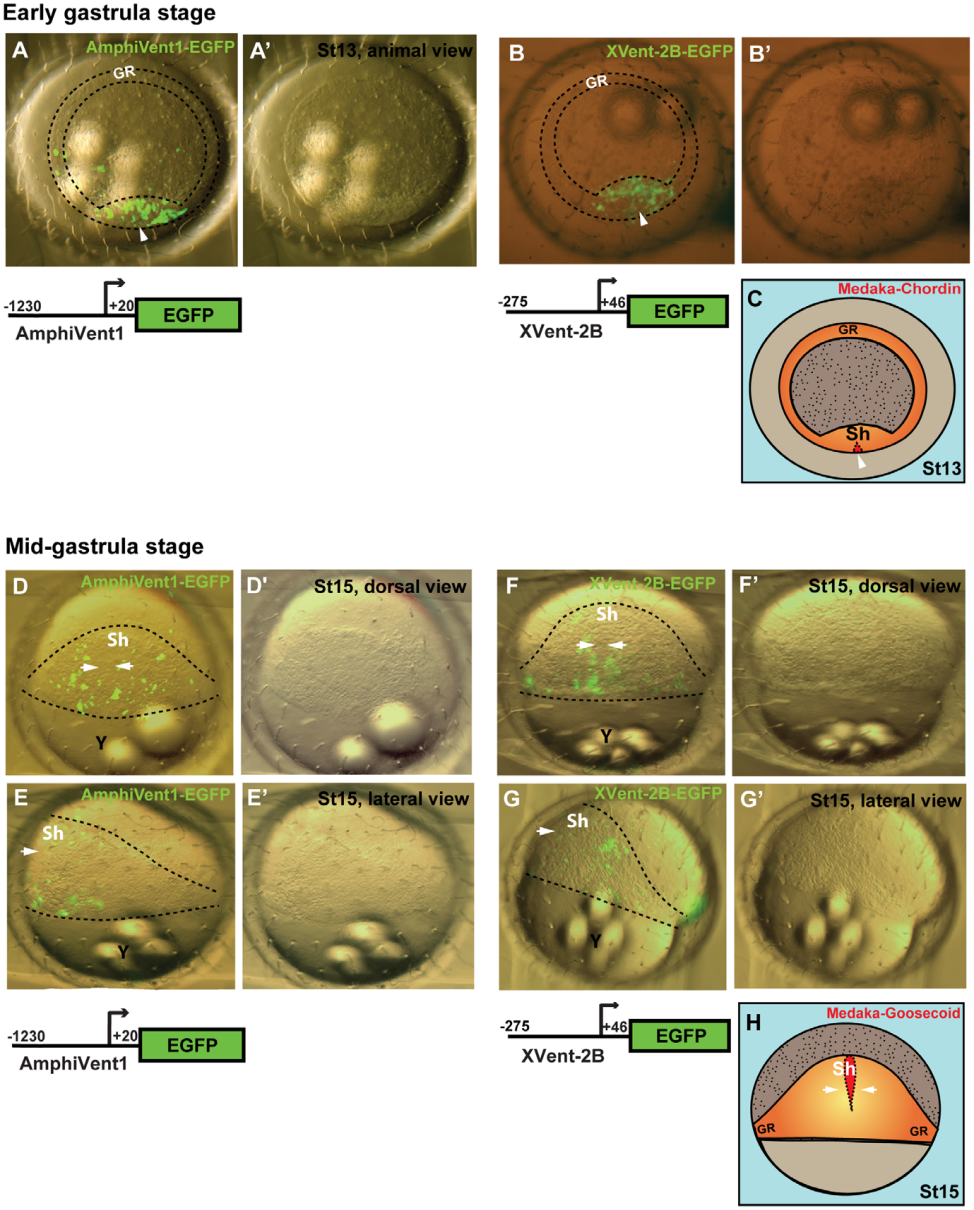
Regulatory potential of *AmphiVent1* and *Xvent-2B* promoters in early developing medaka embryo. Transient expression of EGFP in medaka embryos injected with p817-AmphiVent1 (A-A′, D-E′) and p817-Xvent-2B (B-B′, F-G′) constructs examined under bright field (A′, B′, D′, E′, F′, G′) and bright field merged with fluorescence (A, B, D, E, F, G). (A-B′) EGFP expression driven by *AmphiVent1* or *Xvent-2B* promoter at early gastrula stage. (D-G′) EGFP expression patterns in mid-gastrula stage medaka embryos injected with p817-AmphiVent1 (D-E′) and p817-Xvent-2B (F-G′); dorsal (D-D′, F-F′) and lateral (E-E′, G-G′) views show EGFP fluorescence in the blastoderm around the most dorsal region of embryonic shield (Sh). (C, H) Schematic diagram of developing medaka embryo depicting mRNA expression pattern of early dorsal mesoderm markers *Chordin* (stage 13, C) [30] and *Goosecoid* (stage 15, H) [31]. Dashed line indicates the borders of the blastoderm. White arrowheads depict the most dorsal embryonic shield of the medaka embryo, where *Chordin* and *Goosecoid* are expressed. GR-germ ring, Sh-embryonic shield.

We next asked whether the amphioxus *AmphiVent1* (1.2 kb), *AmphiVent2* (0.9 kb) and *Xenopus Xvent-2B* (0.3 kb) promoters are activated in medaka embryos. Corresponding EGFP reporter constructs p817-AmphiVent1, p817-AmphiVent2 and p817-Xvent-2B were injected into medaka embryos at the single cell stage and their transient expression was monitored during early embryogenesis. At early gastrula stage *AmphiVent1*(Fig. 6A-A′), *Xvent-2B* (Fig. 6B-B′), and *AmphiVent2* (Fig. S3A-A′) promoters were activated throughout the dorsal blastoderm of the embryo, demarcating the region of the most dorsal embryonic shield, where dorsal mesodermal marker *Chordin* is expressed (Fig. 6C) [30]. The EGFP signal from the p817-AmphiVent1 (Fig. 6D-E′) and p817-AmphiVent2 (Fig. S3B-C′) constructs remained evident during gastrulation and its pattern resembled the activation of EGFP in the embryos injected with p817-Xvent-2B (Fig. 6 F-G′). At mid-gastrula stage the strongest EGFP signal was observed laterally from growing embryonic shield. Neither *AmphiVent1* nor *Xvent-2B* promoter was activated in the area of the embryonic shield, where dorsal mesodermal marker *Goosecoid* is expressed in the medaka embryo (Fig. 6H) [31]. We detected ventrolateral expression of EGFP driven by the *Xvent-2B* promoter (Fig. 6G). In contrast, the *AmphiVent1* promoter was not activated ventrally in medaka embryo at mid-gastrula stage (Fig. 6E). It is interesting to note that, in contrast to the *Xvent-2B* promoter, *AmphiVent1* and *AmphiVent2* promoters were activated dorsolaterally, but not ventrally, in the medaka embryo. These observations are in agreement with known expression pattern data for vertebrate and cephalochordate Vent genes. The expression of *Xenopus* vent genes and *AmphiVent1* was demonstrated to be highly similar in dorsolateral but not ventrolateral mesoderm [3]. Whereas somites and their derivatives originate from dorsolateral mesoderm, ventrolateral mesoderm gives rise to the heart and other components of circulatory system, which is generally much simpler in cephalochordates than in vertebrates.

Taken together, our data suggest that the *AmphiVent1* promoter is correctly regulated in the vertebrate embryo and that its spatiotemporal activity is by large similar to the activity of *Xvent-2B* promoter in the same context.

### All Vent proteins function as transcriptional repressors and interact with groucho co-repressors

**Figure 7:**
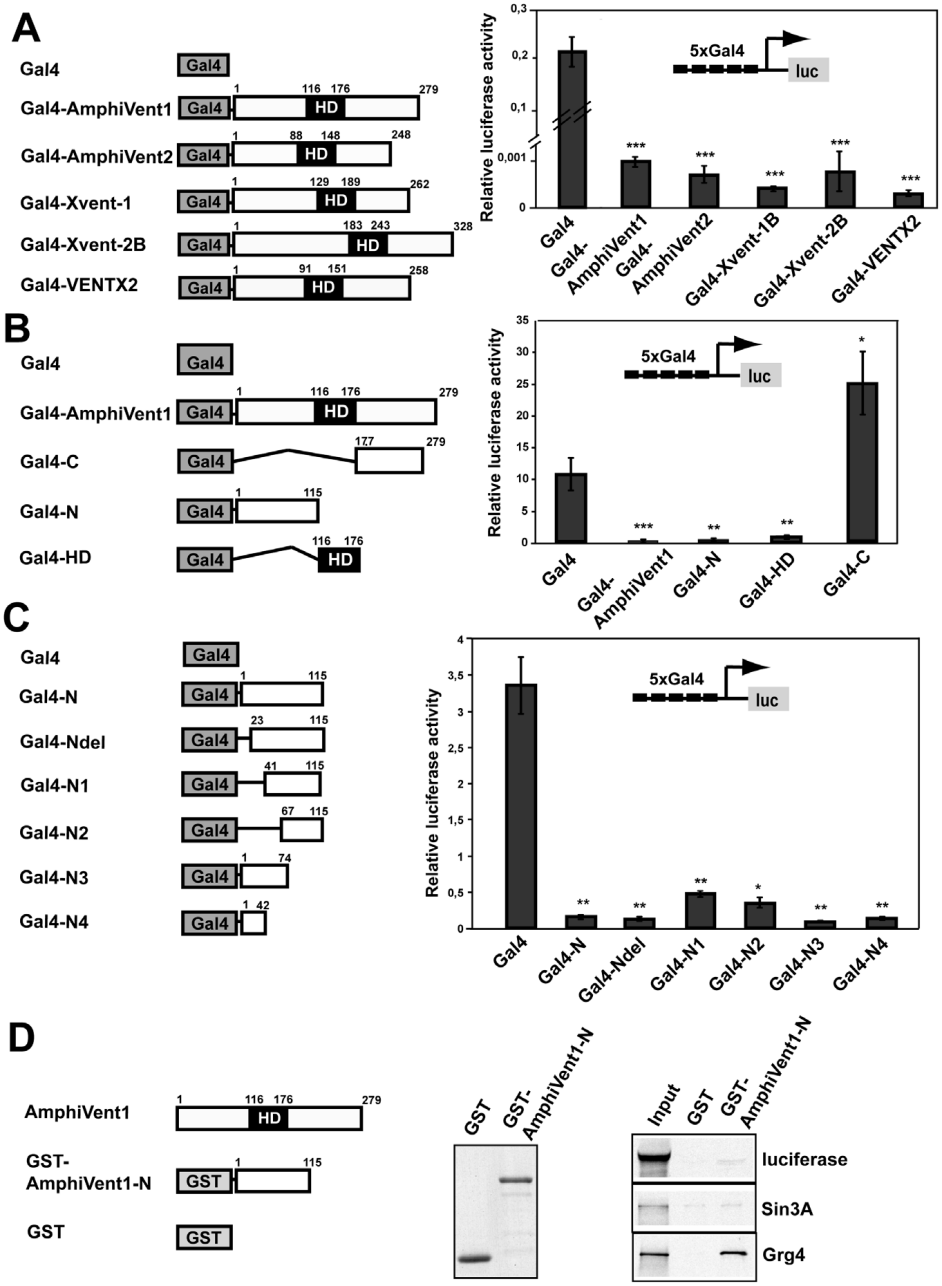
AmphiVent1 is a potent transcriptional repressor and interacts with co-repressor Grg4. (A) The expression plasmids encoding Gal4, Gal4-AmphiVent1, Gal4-AmphiVent-2, Gal4-Xvent1B, Gal4-Xvent-2B were cotransfected with a Gal4 reporter plasmid into 293T cells. (B, C) Expression plasmids encoding Gal4 fusions with various domains of AmphiVent1 were cotransfected with Gal4 reporter plasmid into 293T cells. (D) The N-terminal repression domain of AmphiVent1 interacts with co-repressor Grg4. Schematic diagram of the construct is shown to the left. GST or GST-AmphiVent1-N proteins were bound to Glutathione-Sepharose beads and analyzed by SDS PAGE (middle panel). Please, note equal levels of protein loaded onto beads. GST pull-down assay with *in vitro* produced S^35^-labelled luciferase (negative control), Sin3A and Grg4 co-repressor. Input represents 10% of *in vitro* synthesized proteins used for binding assay. Note that only Grg4 efficiently interacts with the AmphiVent1 N-terminal repression domain. *P<0.05, **P<0.01, ***P<0.001.

*Xenopus Xvent-2B* and zebrafish Vent genes were shown to act as transcriptional repressors [4], [6], [11]. Besides, activating function of Xvent-2 was described [15], [32]. To examine the transcriptional properties of chordate vent proteins, a Gal4 reporter assay was employed. Plasmids encoding Gal4 fusions with AmphiVent1, AmphiVent2, Xvent-1b, Xvent-2b and VENTX2 were cotransfected with Gal4-dependent reporter gene. As shown in Fig. 7A, all vent proteins strongly repressed expression of the reporter gene when tethered to the promoter via Gal4 binding sites. We further focused on identifying the functional domains within AmphiVent1 that mediate transcriptional repression. AmphiVent1 and AmphiVent2 show high amino acid sequence homology within the entire open reading frame (80% identity, 84% similarity) suggesting similar molecular properties. From the two amphioxus vent proteins we selected AmphiVent1 since it is encoded by a previously characterized gene that represents an important marker of ventral mesoderm in amphioxus [2], [3]. To this end, Gal4 fusion constructs encoding different domains of AmphiVent1 were cotransfected together with the Gal4-dependent reporter plasmid into 293T cells. The Gal4 fusion proteins containing the N-terminus or the homeodomain repressed transcription 21-fold and 10-fold, respectively (Fig. 7B). In contrast, the Gal4 fusion protein containing the C-terminus of AmphiVent1 activated transcription 2.2-fold (Fig. 7B). These results suggest that overall, AmphiVent1 acts as a transcriptional repressor and has strong repression domains at its N-terminus and within the homeodomain. In addition, there is a weak transcriptional activation domain located within the C-terminus of AmphiVent1 like in *Xenopus* Xvent-2B protein [15]. Next, we tried to identify specific amino acid sequences within the N-terminus which are responsible for the repression function of AmphiVent1. We cotransfected Gal4 fusion constructs encoding AmphiVent1 amino acids 1-116, 23-116, 41-116, 67-116, 1-74 and 1-42, respectively. All fusion proteins strongly repressed expression of the reporter gene (Fig. 7C). These data suggest that the AmphiVent1 protein likely has multiple independent repressor domains within its N-terminus. Sin3A and Groucho family members appear to be widely used cofactors mediating transcriptional repression of many DNA-binding proteins, including those containing a homeodomain [33], [34], [35], [36], [37]. We therefore tested a possible interaction of the AmphiVent1 N-terminal domain with these obligatory co-repressors. As shown in Fig. 7D, the AmphiVent1 N-terminal domain is able to interact with mouse Grg4 but not with human Sin3A. Interaction of AmphiVent1 with groucho-like co-repressors may be responsible, at least in part, for function of AmphiVent1 as a potent transcriptional repressor.

### AmphiVent1 transcription factor represses AmphiGoosecoid and AmphiChordin promoters

**Figure 8:**
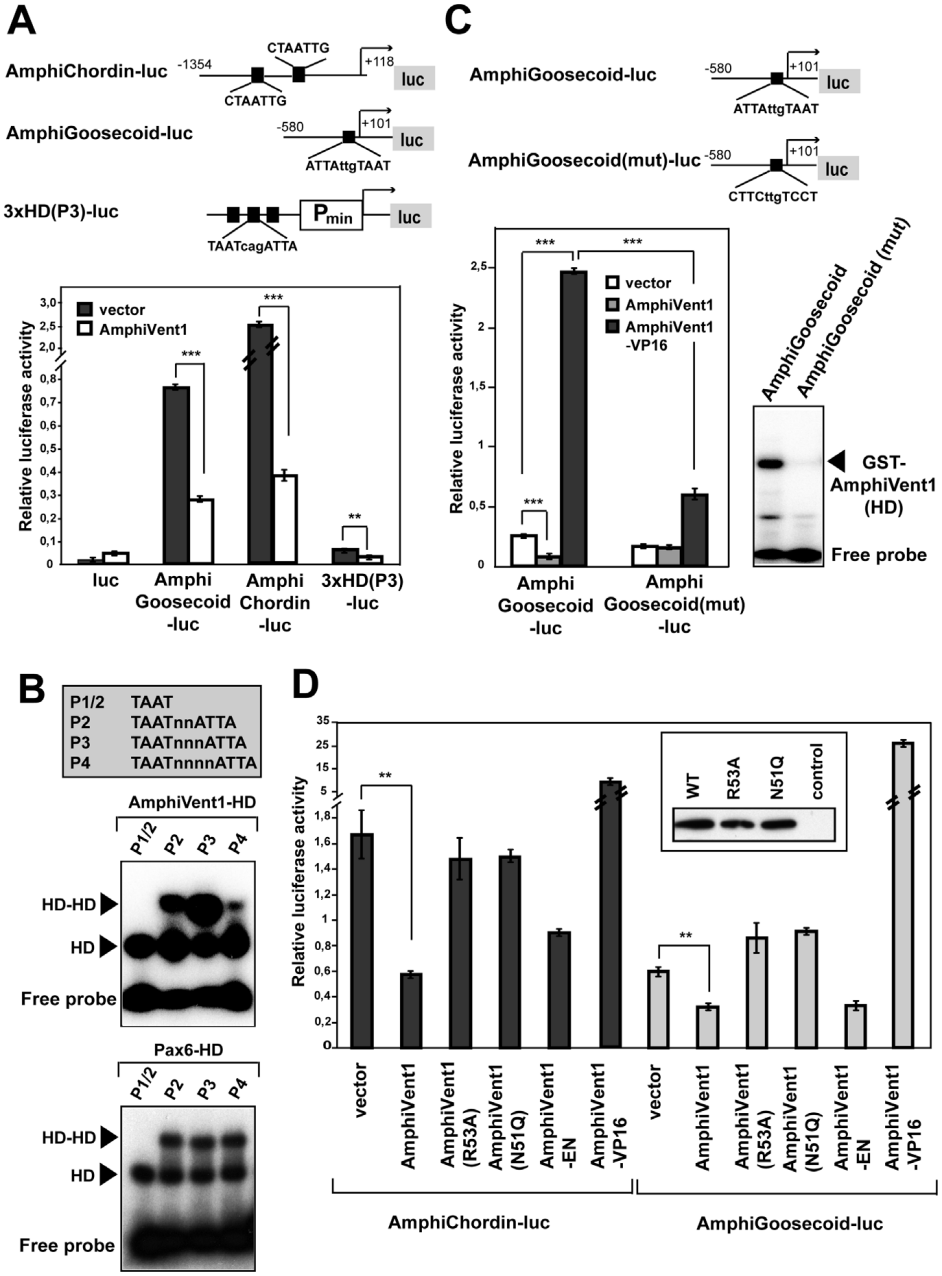
*AmphiChordin* and A*mphiGoosecoid* promoters are directly regulated by AmphiVent1. (A) AmphiVent1 represses *AmphiChordin* and *AmphiGoosecoid* promoters. Schematic diagram of reporter gene constructs with putative AmphiVent1 binding sites in *AmphiGoosecoid* and *AmphiChordin* promoters marked by black boxes (top). Reporter genes AmphiChordin-luc, AmphiGoosecoid-luc and 3xHD(P3)-luc (synthetic homeodomain-responsive reporter gene) were cotransfected into 293T cells with or without AmphiVent1 expression vector (bottom). (B) EMSA of AmphiVent1 and Pax6 (control) homeodomains with binding sites shown in grey shaded box. (C) Schematic diagram of *AmphiGoosecoid* reporter genes with the sequence of wild-type and mutated homeodomain binding site indicated (top). Luciferase reporters containing wild-type and mutated AmphiGoosecoid promoter were cotransfected with or without plasmids encoding AmphiVent1 or AmphiVent1-VP16 fusion protein (bottom, left panel). The effect of *AmphiGoosecoid* promoter mutation on AmphiVent1 binding is documented by EMSA (bottom, right panel). (D) AmphiChordin-luc and AmphiGossecoid-luc reporters were cotransfected with or without expression plasmids encoding wild-type AmphiVent1, DNA-binding deficient AmphiVent1 (R53A, N51Q), AmphiVent1-EN (artificial repression construct) and AmphiVent1-VP16 (artificial activation construct). Note that only the wild-type, but not the DNA-binding mutants of AmphiVent1 can repress the reporter genes. Equal expression of wild-type and mutant AmphiVent1 proteins is documented by Western blotting using antibody against the N-terminally engineered FLAG epitope (inset). Please, note that as predicted from structure-function analysis AmphiVent1-EN functions as wild-type AmphiVent1 (mediates repression), whereas AmphiVent1-VP16 mediates an opposite effect (strong activation of reporter genes). **P<0.01, ***P<0.001.

One of the most prominent functions of vertebrate Vent transcription factors is repression of organizer-specific genes such as *Goosecoid* and *chordin*. To analyze whether AmphiVent1 acts as transcriptional repressor of *AmphiGoosecoid* and *AmphiChordin* genes, we first generated luciferase reporters containing *AmphiGoosecoid* (−580/+101) and *AmphiChordin* (−1354/+118) promoter regions. Cotransfections of AmphiGoosecoid-luc and AmphiChordin-luc plasmids with an expression vector carrying the AmphiVent1 coding sequence resulted in downregulation of reporter genes 2.8-fold and 6.8-fold, respectively (Fig. 8A). Paired-type homeodomains interact with a core TAAT motif as monomers or as homo/heterodimers with dimer sites containing inverted TAAT core motifs separated by several nucleotides [38], [39], [40], [41]. A synthetic homeodomain reporter gene designated 3xHD(P3)-luc that contains three palindromic homeodomain binding sites (TAATcagATTA) was repressed 2-fold by AmphiVent1 (Fig. 8A). In case of 3×HD(P3)-luc the two TAAT core motifs are separated by three nucleotides (P3). In order to better define the DNA-binding specificity of AmphiVent1 and spacing requirements, we performed EMSA with AmphiVent1 homeodomain and a series of binding sites (Fig. 8B). Although AmphiVent1 homeodomain was able to interact with a single TAAT motif in the P1/2 binding site, homeodomain dimerization was observed on P2-P4 binding sites. In comparison with a related paired-type homeodomain of Pax6, AmphiVent1 has a conspicuous preference for a three-nucleotide spacer (P3) (Fig. 8B). It was shown previously that the sequence CTAATTG is critical for Xvent-2B binding, and that the binding is enhanced by the presence of an additional ATTA motif six or seven nucleotides 3′ of the core TAAT [6]. In addition to multiple TAAT core motifs we found two CTAATTG motifs in *AmphiChordin* promoter at positions −574/−580 and −687/−682 (Fig. 8A and data not shown). The CTAATTG motif is not present in *AmphiGoosecoid* promoter, but instead, we identified a putative P3-like palindromic homeodomain-binding sequence ATTAttgTAAT at a position −56/−68. To investigate whether AmphiVent1 is able to repress *AmphiGoosecoid* promoter through this binding site, *AmphiGoosecoid* promoter-containing reporter plasmid with a mutated homeodomain motif (CTTCttgTCCT) was generated and designated AmphiGoosecoid(mut)-luc (Fig. 8C). Binding of the AmphiVent1 homeodomain protein in EMSA was readily detectable to the wild-type *AmphiGoosecoid* promoter sequence, but not to its mutated version (Fig. 8C, lower right panel). In accordance with this data, AmphiVent1 was not able to repress the AmphiGoosecoid(mut)-luc reporter gene (Fig. 8C, lower left panel). To further corroborate our results we converted AmphiVent1 to an activator by fusing it to a strong transactivation domain derived from the VP16 transcription factor. As shown in Fig. 8C, AmphiVent1-VP16 was able to strongly activate the wild-type AmphiGoosecoid-luc reporter gene, but not the AmphiGoosecoid(mut)-luc in which the homeodomain binding site was mutated. Combined, these data suggest that repression of the *AmphiGoosecoid* promoter by AmphiVent1 is mediated via P3-like binding site. Likewise, AmphiVent1-VP16 was able to strongly activate the AmphiChordin-luc reporter gene, whereas fusion of AmphiVent1 to the engrailed repression domain (AmphiVent1-EN) generated a transcription factor with properties comparable to wild-type AmphiVent1 (Fig. 8D). It is well established that the homeodomain can both bind DNA and mediate protein-protein interactions [42]. To investigate whether the DNA-binding function of AmphiVent1 homeodomain is critical for the downregulation of both *AmphiGoosecoid* and *AmphiChordin* genes, we generated two mutants, AmphiVent1(R53A) and AmphiVent1(N51Q), respectively, that contain point mutations in the DNA-binding helix of the homeodomain. Based on previous report [43] these mutations are predicted to disrupt DNA-binding ability of the paired-type homeodomain such as AmphiVent1. AmphiVent1(R53A) and AmphiVent1(N51Q) proteins were no longer able to repress *AmphiGoosecoid* and *AmphiChordin* reporter genes (Fig. 8D) despite normal level of expression (Fig. 8D, inset). Taken together, our data show that the AmphiVent1 homeodomain protein is able to act as a direct transcriptional repressor of *AmphiGoosecoid* and *AmphiChordin* genes.

## Discussion

Orthologous genes encoding components of Bmp signaling and transcription factors downstream of Bmp signaling (such as Vent and Goosecoid) are expressed in highly similar patterns in vertebrates and a cephalochordate amphioxus [2]. However, the key question is whether the conservation of expression patterns of network constituents necessarily means conservation of functional network interactions. Furthermore, even if part (a core) of the gene regulatory network is evolutionarily conserved, one has to explain how an increased morphological complexity is achieved.

Here, we performed a detailed molecular analysis of gene regulatory network underlying DV patterning in a basal chordate amphioxus. Although we believe that the data presented in this study are relevant for amphioxus it must be emphasized that most of the experiments were performed *in vitro* using heterologous cell lines and heterologous proteins or heterologous animal model system (medaka) which may induce a bias in the results and influence our interpretation. In addition, since no data on transgenic amphioxus are available for *AmphiVent1* or *AmphiVent2* promoter constructs used in this study, we do not know whether we have isolated sufficiently large promoter fragments that can account for the entire expression domain of these two genes.

### Vent genes in chordate evolution: from indispensableness to damnation

Genetic and molecular studies have identified a remarkably conserved function of the Bmp-Chordin signaling network in animal DV patterning [1]. The system, which relies on production of secreted Bmp inhibitors such as Chordin, antagonizing the activity of Bmps with resulting gradient of Bmp activity along the DV axis, seems to be utilized in invertebrates as well as in vertebrates [1], [2], [44]. However, an impressive conservation of the key players (Bmp and Chordin) does not eventuate in the conservation of all other components in the network. For example, Vent genes, which are critically important for DV patterning in ‘lower’ vertebrates [4], [7], [11], [12] and cephalochordates (this study), are apparently dispensable for the Bmp-Chordin signaling network in mammals [45] (this study). It is interesting to note that the number of Vent-like genes differs among the chordate species. Amphioxus genome contains two Vent genes copies (*AmphiVent1* and *AmphiVent2*) [10], which are situated on the same chromosome in close proximity and have likely arosen by a lineage-specific duplication. Three and four Vent-like genes are present in the genome of zebrafish and *Xenopus*, respectively. A single Vent gene (*VENTX*) is present in the genome of humans and chimpanzee. In amphibians and teleosts mutational analysis of Vent genes suggested their prominent role in DV patterning [4], [9], [16]. Products of zebrafish *Vent*, *Vox*, *Ved* and *Xenopus Xvent-1*/*Xvent1b* and *Xvent-2*/*Xvent2b* genes are homeodomain-containing repressor proteins, which at early stages of development suppress organizer-specific genes Goosecoid and Chordin [4], [7], [9], [11], [46]. It was shown that *Xenopus Goosecoid* promoter region contains the Xvent-2 binding site and is directly repressed by Xvent-2 [6], [9]. In our study we have provided evidence for direct repression of amphioxus *Goosecoid* and *Chordin* gene promoters by the AmphiVent1 protein. However, it appears that in mammals Vent genes have completely abandoned their prominent function within the Bmp-Chordin signaling network [18] (this study). First of all, a functional copy of Vent gene has been apparently lost in the mouse genome. We have identified a sequence encoding a remnant of a homeodomain homologous to exon2 and exon3 of the human *VENTX* gene that is located on mouse chromosome 7 between *Utf1* and *Adam8*. This region precisely corresponds to a syntenic region where human orthologue *VENTX* is situated on human chromosome 10. Moreover, the functional role of *VENTX* is not clear. We have shown that in contrast to *AmphiVent1/2* and *Xvent1/2* promoters, the promoter of *VENTX* is not Bmp-inducible. Expression of the *VENTX* gene has only been detected in immature bone marrow, indicating that the gene may be involved in the maintenance of hematopoietic stem cells in the adult [45]. Its expression is downregulated in lymphocytic leukemias, suggesting a potential role of *VENTX* in the clinical behavior of hematopoietic malignancies [47]. Structure-function studies have shown that the human VENTX2 protein functions as a transcriptional repressor [45] (this study), a feature conserved among other Vent proteins (this study). Functional equivalence of human VENTX and zebrafish/*Xenopus* Vent proteins was confirmed by microinjection of VENTX mRNA that resulted in ventralized zebrafish embryo [45]. Recently, a new protein partner for VENTX and Xvent-2 has been identified. Gao et al. have reported that Xvent-2 and VENTX are Lef/Tcf-associated factors [47], [48]. However, their interaction with Lef/Tcf results in distinct functional properties. Upon association with Lef/Tcf *Xenopus* Xvent-2 seems to be an activator, whereas human VENTX protein functions as a suppressor of canonical Wnt signaling. Gao et al. proposed that Xvent-2 interacts with Tcf/Lef directly through its homeodomain and activates gene expression by the N-terminal transactivation domain [47]. More recently, Gao et al. argue that the mechanism of transcriptional repression by VENTX is caused by the disruption of the complex between β-catenin and TCF/LEF factors [48]. Using reporter gene assays we have been able to reproduce transactivation results for vertebrate Vent proteins reported previously [47], [48], i.e. transcriptional stimulation in the case of XVent-2 and suppression for VENTX (Fig. S4). However, in the same transcriptional assay AmphiVent1 was not able to influence the Lef/Tcf-responsive reporter gene (Fig. S4) despite high sequence conservation of AmhiVent1, Xvent-2 and VENTX homeodomains (Fig. 1A), which are the presumed interaction domains. One obvious explanation is the lack of a strong transactivation domain in AmphiVent1. This is unlikely since the fusion of AmphiVent1 with strong transcriptional activator VP16 does not lead to detectable activation of the Lef/Tcf reporter gene (Fig. S4). The molecular mechanism of Vent-mediated modulation of canonical Wnt signaling thus remains unclear.

In conclusion, genome analyses, structure-function and developmental studies point to a dynamic evolution of vent genes. The loss of a presumed ancestral role of Vent genes in chordate DV patterning is particularly intriguing. One plausible explanation of such an impressive damnation of Vent genes in mammals lies probably in marked evolutionary changes in early development of chordates. Sanders and co-workers [18] argue that the lack of Vent gene in the mouse is due to slower development of the mouse embryo. Besides, at very early stages of development mammalian embryo does not need to be polarized for future proper development. In contrast, molecular asymmetry of teleost and amphibian eggs is an inherent condition of their normal development. Many maternal and newly expressed zygotic genes are involved in the highly organized scenario driving the polarization of zebrafish and *Xenopus* embryo. Thus, Vent genes have likely been recruited as one of the key players in this complicated system of molecular asymmetry.

### Conserved BMP regulation of chordate Vent genes

**Figure 9:**
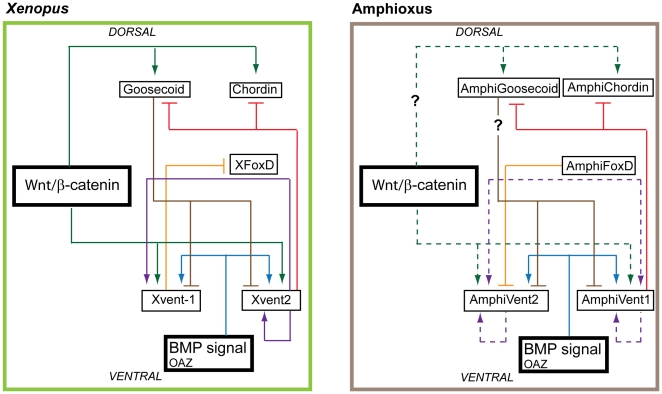
Gene regulatory network (GRN) architecture for early D/V mesoderm specification in *Xenopus* and amphioxus. Experimental data suggest high conservation of Bmp-Chordin signaling network in all chordates. In contrast, the role of Wnt/β-catenin signaling in D/V mesoderm specification seems to be limited to vertebrates. Dashed line indicates interactions, that are present in *Xenopus* but were shown to be absent in Amphioxus GRN (this study). The question mark within the continuous line denotes presumed but not yet proven regulatory link. The question mark within the dashed line denotes likely absence of the regulatory link in amphioxus.

Yu et al. have shown that the expression patterns of Bmp signaling components and its modulators such as Chordin, ADMP, *Goosecoid*, BAMBI, *Tsg* display extreme similarity between amphioxus and vertebrate embryos at early stage of development [2]. Amphioxus embryos treated with BMP4 have a phenotype similar to that resulting from overexpression of Bmp2 and Bmp4 in vertebrate embryos [49]. Exogenous Bmp protein treatment repressed expression of markers of the dorsal mesoderm and caused ventralization of the amphioxus embryo [2]. In early vertebrate (*Xenopus* and zebrafish) embryo Bmp signaling is mediated by Vent genes [4], [11]. Detailed molecular analysis of *Xvent-2* promoter has revealed BRE in its proximal region [12]. The proximal promoter of *Xvent-2* contains Smad and OAZ binding sites, which are known mediators of Bmp4 signaling. Indeed, it was shown that OAZ is able to form a complex with Smad1 and Smad4 upon Bmp stimulation [13]. Furthermore, a cooperative binding of OAZ and Smads to the BRE of *Xvent-2* gene promoter leads to promoter activation. Our data support a concept of a highly conserved role of Bmp regulation in the establishment of D/V axis in chordates. Bmp signaling proteins activate amphioxus *AmphiVent1* and *AmphiVent2* genes. Detailed analysis of the regulation of *AmphiVent1* promoter region has revealed that the activation is mediated by Smad1/Smad4 proteins. Furthermore, zinc finger protein OAZ seems to be involved in the activation of *AmphiVent1* in a similar way as shown previously for the *Xenopus Xvent-2* gene. There is a notable difference in the promoter structure between cephalochordates and amphibian genes with regard to positioning and significance of individual SMAD binding motifs. BRE of the *Xenopus Xvent-2* gene is constrained within a short promoter region between nucleotides −243 to −191 [13]. This region contains a single Smad binding site, which is crucial for Bmp inducibility of *Xvent-2* promoter. In contrast, we have identified six putative Smad binding elements (SBE) in the Bmp inducible region of *AmphiVent1* gene promoter between nucleotides −1230 to +20. Deletion and mutational analysis has revealed that none of these SBE's plays a dominant role in the activation of the gene by Bmp signaling since point mutations of individual SBE's did not lead to the complete loss of Bmp inducibility. Bmp-mediated activation was abolished only in the case of simultaneous mutation of all six SBE's within the −1230 to +20 promoter region of *AmphiVent1*. Therefore, the significance of six SBE's within the *AmphiVent1* promoter region appears to be equally balanced. Seemingly this fact is not even influenced by the presence of OAZ binding site in the vicinity of one of the SBE's. It is of note that the human *VENTX* gene promoter contains a putative SBE, which however does not provide any detectable Bmp inducibility [50] (this study). Our observations provide evidence for a prominent robustness of the cephalochordate *AmphiVent1* promoter with regard to Bmp responsiveness through multiplication of SBE's. In contrast, the evolution of vertebrate Vent gene regulation proceeded by a distinct mechanism. The apparent robustness of Bmp-mediated regulation of *Xenopus* Vent genes was achieved by co-option of Vent binding sites into their promoters. These sites provide a positive autoregulatory loop (Fig. 9), which maintains the Bmp inducibility of *Xenopus* Vent promoters. We have not found any evidence for such an autoregulatory loop in the case of *AmphiVent1* and *AmphiVent2* promoters (Fig. S5). Summarized, our data suggest that BRE-containing cis-regulatory sequences for Smad and OAZ transcription factors were likely present in Vent-like homeobox genes of a chordate common ancestor. Taking into account our data, which indicate the ability of amphioxus Vent proteins to directly repress *AmphiChordin* and *AmphiGoosecoid*, we propose a deep evolutionary conservation of a Bmp-mediated regulatory module within the gene regulatory network (GRN) controlling chordate DV patterning (Fig. 9).

In addition to the transcriptionally-based gene regulatory network, experimental analyses have recently revealed another level of Bmp-mediated regulation via formation of a gradient in *Xenopus* embryo [51]. It was shown that the Bmp activity gradient is defined by a‘shuttling-based’ mechanism, whereby the Bmp ligands are translocated ventrally through their association with Bmp inhibitor Chordin. This mechanism, which may function in amphioxus as well, represents another way to achieve the robustness of Bmp gradient [52].

### The role of Wnt-signaling in the formation of chordate DV axis is not conserved

One of the earliest asymmetrical molecular signals in the developing vertebrate embryo is the nuclear localization of β-catenin on the dorsal side. The β-catenin signal induces the expression of Bmp antagonists such as Chordin and Noggin in dorsal mesoderm (Spemann organizer) of early *Xenopus* embryo [53]. Likewise, in zebrafish, cells of the embryonic shield accumulate β-catenin and thus acquire organizing activity [54]. A significant role of canonical Wnt signaling in the formation of an organizer has not been observed in cephalochordates. Although during early amphioxus development nuclear β-catenin and some Wnts are present around the blastopore [2], overactivation of Wnt signaling by treatment of amphioxus embryos with Li^+^ at early stages of development had no effect on the DV axis specification [17].

Loss-of-function experiments indicate an important role of canonical Wnt signaling in the regulation of Vent genes and specification of vertebrate ventral mesoderm [14], [55]. Moreover, Wnt8 was shown to be able to activate *Xenopus Xvent-2* and *Xvent-1* genes as well as zebrafish *Vent* and *Vox* [14], [15], [56]. Using luciferase reporter assays we have confirmed positive regulation of *Xvent-1* and *Xvent-2* by canonical Wnt signaling (this study, Fig. 4B). It is established that canonical Wnt signaling plays an important role in early mesoderm formation of mammals [57]. We have found that the promoter of the human *VENTX* gene, likely due to its rapid evolution (see above), is not responsive to canonical Wnt signaling. Taking into account an apparent loss of the mouse Vent gene, the available data point to Vent-independent formation of mesoderm in mammals.

It seems that Wnt/β-catenin signaling plays a dual role in the regulation of *Xenopus* and zebrafish Vent genes. In addition to stimulation triggered by Wnt8, β-catenin participates in the suppression of vertebrate Vent genes in dorsal lip at early stages of development. The downregulation is achieved by β-catenin-dependent activation of dorsal-lip-specific transcription factors such as goosecoid, which in turn represses Vent gene expression [7], [18], [58]. Expression data indicate mutually exclusive patterns of *AmphiGoosecoid* and *AmphiVent1* genes in early amphioxus embryo, which makes the above-mentioned mechanism plausible in amphioxus as well [2].

We were interested in whether the Wnt-mediated regulation of Vent genes is an evolutionarily conserved feature between cephalochordates and ‘lower’ vertebrates. It was shown previously that the expression patterns of *Wnt8* and *AmphiVent1* overlap in early amphioxus embryo [2]. At a mid-gastrula stage *AmphiVent1* and *Wnt8* are co-expressed dorsolaterally in regions of invaginated mesendoderm, which constitute the prospective paraxial mesoderm [2], [3], [59]. Although we have identified putative Lef/Tcf binding sites in *AmphiVent1* and *AmphiVent2* gene promoters by *in silico* analysis, our experimental data *in vitro* (reporter assays) and *in vivo* (pharmacological treatment of embryos) do not indicate that the promoters are regulated by canonical Wnt signaling, which is congruent with results obtained by Li+ treatment that has not impact on DV patterning in amphioxus-[17].

It is interesting to note that canonical Wnt signaling is broadly used for the establishment of anteroposterior (A/P) axis in diverse deutorostomes (including frogs, fish, mammals, birds, amphioxus, and echinoderms) and protostomes [60]. In A/P patterning Wnts are important posteriorizing factors, which influence development of multiple tissues. In contrast, the main outcome of DV polarity specification in chordates is the formation of distinct types of mesoderm from which ventral mesoderm of vertebrates gives rise to a large number of derivatives (such as complicated circulatory system). Co-option of a new signaling pathway (Wnt) in mesoderm patterning might be explained by a need for increased complexity and quantity of ventral mesoderm in vertebrate development. For example, vertebrate embryos need to produce an efficient circulatory system without delay. In contrast, the embryo of amphioxus is very small and does not need to deploy a large amount of mesoderm early to set the stage for rapid production of a highly efficient circulatory system [3].

Summarized, the available experimental evidence indicates that the canonical Wnt signaling pathway does not play a role in the establishment of ventral fate in cephalochordate amphioxus (Fig. 9). This is in principle consistent with a secondary loss of an ancestral feature (Wnt-mediated regulation) in cephalochordates. We, however, favor the hypothesis that the co-option of canonical Wnt signaling pathway for the establishment of DV patterning in vertebrates represents one of the innovations through which an increased morphological complexity of vertebrate embryo is achieved.

### Conclusion

In conclusion, our data show that there is a remarkably conserved gene regulatory network in which Bmp signaling induces transcriptional repressor Vent, which in turn represses genes encoding transcription factor Goosecoid and signaling molecule Chordin. We propose that this gene regulatory network was a key module recruited in early chordate evolution for establishment of DV patterning. This network remains in use in the present-day chordates such as amphioxus, fish and amphibians.

## Materials and Methods

### Ethics statement

Housing of animals and *in vivo* experiments were performed after approval by the Animal Care Committee of the Institute of Molecular Genetics (study ID#36/2007) and in compliance with national and institutional guidelines (ID#12135/2010-17210).

### Bioinformatic tools and phylogenetic analysis

For phylogenetic analysis, we aligned sequences and generated neighbor-joining trees with bootstrap with ClustalX and the GeneDoc program. To construct a Vent tree, we used the following organisms (with accession numbers): Human VENTX2 (AF068006), Pan troglodytes Vent (XP_521666), zebrafish Vox (AF255045), zebrafish Vent (AF255044), Xenopus Xvent-2B (AJ131095), *Xenopus* Xvent-2 (X98849), *Xenopus* Xvent-1B (AJ131094), *Xenopus* Xvent-1 (X92851), amphioxus AmphiVent2 (gene model estExt_gwp.C_7770002; protein ID 289443; http://genome.jgi-psf.org), amphioxus AmphiVent1 (AAK58840), Hs six2 (AF332196). Promoter alignments in Fig. 5 were generated using Family Relations software (http://family.caltech.edu) [61] with a 20 bp window sliding in 1 bp increments.

### Plasmids

The amphioxus amphiVent1, amphiVent2, amphiGoosecoid, amphiChordin, xenopus XVent2b and human VENTX2 promoters were amplified by PCR using a corresponding genomic DNA as a template. The oligonucleotides for PCR were as follows (5′-3′): AmphiVent1: forward 616A, ATCATGAATGAATAACAATGACGTTG; reverse 616B, GTTGTCGCGTGTTCGTCACTGGA; AmphiVent2: forward 822A, TGAAATTTGTTCGCTTACAGTGTA; reverse 822C, GGTCGACGATTGACAGCAGTG; AmphiGoosecoid: forward 918A, CAATGGGCAGGTTGATAATCCACT; reverse 918B, CGTGACTGTTTCCGCTGCTTTGTC; AmphiChordin: forward 839A, CAGACAACGTCAAAAGACAGTCAA; reverse 839B, TTCAGAGAATGTTTGCGTCGTCAA; Xvent-2b: forward 542A, GAGAGGCTTCCCAATAGCTA; reverse 542B, CTGTATTAGTCCTTGTGTTC; VENTX2: forward 818A, CATCGCCTCTCCATTAACCAG; reverse 818C, GCCAAAGCTGGAGAGGCGCAT. PCR products were cloned into pGL3-basic vector and sequenced. AmphiVent1 promoter 5′ deletion constructs were obtained by PCR using corresponding oligonucleotides. Site-directed mutagenesis of promoter constructs was performed using QuikChange kit (Stratagene). Smad binding sites within amphiVent1 promoter were mutagenized converting the wild-type core of SBE sequence AGAC into AcAt. The wild-type P3 homeodomain binding site ATTATTGTAAT in amphiGoosecoid promoter was mutagenized into cTTcTTGTccT. Tcf/Lef binding sites in AmphiVent1 and AmphiVent2 promoters were mutagenized converting the wild-type CTTTGTT into aggTGTT. Tcf/Lef binding sites in Xvent-1B and Xvent-2B promoters were mutagenized converting the wild-type CTTTGAT into aggTGAT. All constructs were verified by sequencing. The 5xGal4E1b and 3xP3HD-luc constructs were described previously [38]. Canonical Wnt signaling responsive luciferase reporter plasmid pTOPFLASH containing multiple Tcf/Lef1 consensus binding sites, CMV-based expression vectors encoding human LEF1 and N-terminally truncated (stabilized) human β-catenin (β-cateninΔN) were obtained from Dr. V. Korinek [62]. BMP-responsive reporters pTAZ-BRE/D and pTAZ-BRE/P were generated by inserting distal (nucleotides −1214/−669) or proximal (nucleotides −669/−218) region of amphiVent1 promoter into luciferase reporter plasmid pTA (Clontech). Expression vectors encoding human Smad1, Smad4, Smad4-D4, caAlk2, hOAZ were kindly provided by Drs. P. ten Dijke, M. Whitman and J. Massague. To generate a dominant-negative hOAZzf6-13 construct, a region corresponding to zinc fingers 6 to 13 of hOAZ was amplified by PCR and cloned into pKW-Flag. To obtain full-length amphiVent1 constructs, the open reading frame of amphiVent1 was cloned into expression plasmids pKW-Flag, pKW-Flag-VP16, pKW-EN and pCS2. AmphiVent1 DNA-binding mutants R53A and N51Q were generated by QuikChange kit (Stratagene). To obtain full-length Gal4-Vent constructs, the open reading frames of amphiVent1, amphiVent2, Xvent1, Xvent2b (kindly provided by C. Niehrs) and VENTX2 (kindly provided by R. D'Andrea) were amplified from plasmid templates using PCR and cloned into a Gal4 expression plasmid pKW-HA-Gal4. To generate Gal4 fusion constructs containing individual domains of amphiVent1, the corresponding regions of amphiVent1 cDNA were amplified by PCR and cloned into the Gal4 expression plasmid. To generate GST fusion expression plasmid pET42-amphiVent1N, the region N-terminal to a homeodomain was amplified by PCR and cloned into pET42a.

### Cell Culture, Transient Transfection and Luciferase Reporter Assay

293T, MDA-MB468 and P19 cells were purchased from ATCC. 293T and MDA-MB468 cells were cultured in Dulbecco's modified Eagle's medium (SIGMA) supplemented with 10% Fetal bovine serum (PAA laboratories), 2 mM L-glutamine, 100 units/ml penicillin, and 0.1 mg/ml streptomycin (SIGMA). P19 cells were cultured in Dulbecco's modified Eagle's medium (SIGMA) supplemented with 5% Fetal bovine serum (PAA laboratories), 2 mM L-glutamine, 100 units/ml penicillin, and 0.1 mg/ml streptomycin. Cells were passaged every three days and maintained at 37°C in an atmosphere of humidified air with 5% CO2. Cells were plated in 24-well plates 24 hours prior to transfection. Each well was transfected with 100 ng of the reporter gene and 50 ng of the expression vector (when applicable) using Fugene 6 (Roche) according to the manufacturer's protocol. The total amount of DNA transfected per well was 300 ng and was adjusted with pUC18 when necessary. A β-Galactosidase expression plasmid was cotransfected to normalize the transfection efficiency. In some experiments, 24 hours after transfection recombinant human BMP2, BMP4, BMP7, TGFβ or Activin B (all from R&D) was added.Unless indicated otherwise, Bmp signaling was stimulated by the addition of human BMP2 at a final concentration of 20 ng/ml. Alternatively, Bmp pathway stimulation was elicited by transfection of 50 ng of the caAlk2 expression vector. Two days after transfection, the cells were lysed in 100 ul of 1× passive lysis buffer (Promega). Luciferase reporter assays were performed using Luciferase Reporter assay kit (Promega). β-Galactosidase was detected with Galacto-Star system (Applied Biosystems). All transfection experiments were performed at least three times and a representative experiment is shown. In each experiment triplicate assays were performed; graph values represent the average of triplicates +/− standard deviation. Statistical significance was determined using Student t-test in Microsoft Excel.

### Electrophoretic Mobility Shift Assay (EMSA)

The following double-stranded oligonucleotides were used in EMSA with amphiFoxD (5′-3′, only top strand is shown for simplicity): FoxD1 BS 1, CTTAAGTAAACAAACA; FoxD1 BS 2, AGGCCGTAAACAAACA; FoxD1 BS 1(K.O.), CTTAAGTACCCAAACA; FoxD2 BS, CTTAAGTAAACAATGG; FoxC1 BS, CCAAAGTAAATAAACA; FoxC1 BS (K.O.), CCAAAGTAAATTAACA; amphiFoxD BS V1, TCACAGCAAACAATTA; amphiFoxD BS V2, TCAATGTAAACAATAG. The following double-stranded oligonucleotides were used in EMSA with amphiVent1-HD and Pax6-HD (5′-3′, only top strand is shown for simplicity): amphiGoosecoid promoter WT, GCATGCTAAATTATTGTAATGAATGCGCA; amphiGoosecoid promoter mut, GCATGCTAAcTTcTTGTccTGAATGCGCA; P1/2, TCGACTGAGTCTAATTGAGCGTCT; P1, TCGACCCTAATGATTACCCTCGA; P2, TCGACCCTAATCGATTACCCTCGA; P3, TCGACCCTAATGCGATTACCCTCGA; P4, TCGACCCTAATGCGCATTACCCTCGA. Double-stranded oligonucleotides containing indicated homeodomain and FoxD binding sites were radioactively labeled at the 5′ends with γ^32^PdATP using polynucleotide kinase (Boehringer Manheim) and purified on microspin columns (Amersham Biosciences). ^32^P-labeled oligonucleotides were incubated with bacterially-purified 6xHis-tagged or GST- tagged homeodomain proteins in binding buffer (10 mM HEPES at pH 7.7, 75 mM KCl, 2.5 mM MgCl2,0.1 mM EDTA, 1 mM DTT, 20% glycerol, 0.5% BSA, and 0.1 mg/mL poly-dIdC) for 15 minutes at RT. In some experiments increasing amounts of unlabelled double-stranded oligonucleotides were added to binding reaction to test for specificity. Samples were analysed by 6 % polyacrylamide gel electrophoresis and autoradiography.

### GST-Pull Down Assay

^35^S-labeled Grg4 was prepared by TNT Quick Coupled Transcription/Translation Systems according to the manufacturer's protocol (Promega). GST fusion expression plasmids were transformed into BL21 CodonPlus (DE3)-RIPL cells (Stratagene). A single colony from the transformation was cultured in 2 ml LB medium containing 50 µg/ml of chloramphenicol and 30 ng/ml of kanamycin overnight at 37°C. The cultures were transferred to 100 ml of LB without antibiotics. The expression of the fusion construct was induced by adding IPTG to a final concentration of 2 mM for 2 hours. The cells were harvested by centrifugation and resuspended in 5 ml of NETN buffer (20 mM Tris pH 8.0, 100 mM NaCl, 1 mM EDTA, 0.5% NP40). Lysozyme was added to a final concentration of 0.1 mg/ml. The lysates were incubated on ice for 20 min, sonicated and centrifuged to remove the cell debris. The supernatant was incubated with 200 µl of glutathione-Sepharose beads slurry (BD Bioscience) for 1 hour at 4°C. The beads were washed three times by 5 ml of binding buffer (20 mM Tris pH 8.0, 100 mM KCl, 5 mM MgCl2, 0.1 mM EDTA, 20% Glycerol) containing 0.1% NP40. GST fusion proteins bound to the beads were analyzed by SDS-PAGE. Beads containing normalized amounts of fusion proteins were blocked by binding buffer containing 0.05% of NP40 and 5 mg/ml of BSA for 2 hours at 4°C and resuspended in 150 µl of binding buffer containing 0.05% NP40, 1 mg/ml BSA and 100 µg/ml Ethidium Bromide. The beads were incubated overnight at 4°C with 3 µl of ^35^S-labeled Grg4. The beads were washed three times with 500 µl of binding buffer containing 0.05% NP40 and boiled with SDS sample buffer. The amount of Grg4 was detected by autoradiography.

### SceI-mediated transgenesis in medaka fish

*I-SceI* meganuclease transgenesis in medaka fish was performed as previously described [63]. Fertilized eggs of inbred Cab strain were collected immediately after spawing and were placed into cold (4°C) 1 × Yamamoto's embryo rearing medium [64]. One cell stage embryos were injected with the solution containing *I-SceI*-plasmids (AmphiVent1-GFP, XVent-2B-GFP) and 0.25U/µl *I-SceI* meganuclease in 0.5 × *I-SceI* buffer (New England Biolabs, USA)/0.5 × Yamamoto's embryo rearing medium. Final concentration of injected plasmids was 10 ng/µl of AmphiVent1-GFP and 15 ng/µl of XVent-2B-GFP, respectively. The expression of the transgene was detected as early as at an early gastrula stage (St.13) and onward [64]. The injecting setup was as follows: pressure injector Femtojet (Eppendorf); micromanipulator TransferMan NK (Eppendorf); borosilicate glass capillaries (GC100F10, Harward Apparatus); stereomicroscopes (Olympus SZX7, SZX9).

### Embryo treatment, RNA purification and real-time quantitative RT–PCR (qRT–PCR)

Developing embryos of *B. floridae* were collected into RNA later (Ambion). Some embryos treated with DMSO (control) or 3 µM Wnt signaling activator 6-Bromoindirubin-3′-oxime (BIO; Sigma) [23] at blastula stage and allowed to develop until mid-neurula stage. Standard procedures were used for RNA purification and reverse transcription. Briefly, total RNAs were isolated from embryos using the Trizol reagent (Invitrogen); contaminating genomic DNA was eliminated by DNAse I digestion and RNA was repurified using RNeasy Micro kit (Qiagen). Random-primed cDNA was prepared in a 20 µl reaction from 100 ng of total RNA using SuperScript VILO cDNA Synthesis kit (Invitrogen). cDNAs were produced from at least two independent RNA isolations and the PCR reactions were performed in triplicate for each primer set. Control reactions (containing corresponding aliquots from cDNA synthesis reactions that were performed without reverse transcriptase; minus RT controls) were run in parallel. PCR reactions were run using the LightCycler 480 Real-Time PCR System (Roche). Typically, a 10 µl reaction mixture contained 5 µl of LightCycler 480 SYBR Green I Master mix (Roche), 1 µl of primers (final concentration 0.5 µM) and cDNA diluted in 4 µl of deionized water. Crossing-threshold (CT) values were calculated by LightCycler® 480 Software (Roche) using the second-derivative maximum algorithm. The specificity of each PCR product was analysed using the in-built melting curve analysis tool for each DNA product identified; additionally, PCR products were verified by sequencing. All primers were calculated using Primer 3 computer services at http://frodo.wi.mit.edu/. The housekeeping gene encoding ribosomal protein L32, *AmphiRPL32,* was used as internal control gene to standardize the quality of different cDNA preparations. Primer sequences were as follows: *AmphiRPL32* (117 bp product): forward 1093A, GGCTTCAAGAAATTCCTCGTT; reverse 1093B, GATGAGTTTCCTCTTGCGTGA; *AmphiVent1* (204 bp product): forward 1094A, ACGTCTGACGAGGAGGAAGA; reverse 1094B, GTACTTCTGCAGGCGGAAAC; AmphiVent2 (219 bp product): forward 1096A, GACGAGGAGATCGACGTTGT; reverse 1094B, GTACTTCTGCAGGCGGAAAC; *AmphiAxin* (148 bp product): forward 1098A, TCATGTGCTACCCTCCATTTC; reverse 1098B, TCATCCAGTCGTTCCTCATTC; *AmphiFoxQ2* (255 bp product): forward 1103A, TCTACCAGTGGATCATGGACAA; reverse 1103B, CGTACTGCATGTAGGGATGCT.

## Supporting Information

Figure S1 Activation of Amphioxus and Xenopus Vent gene promoters by Bmp2, BMP4 and BMP7. (A) P19 cells were transfected with luciferase reporters containing AmphiVent1, AmphiVent2, Xvent-2B and VENTX2 5′genomic non-coding regions in the absence (open bars) and presence (black bars) of increasing amounts of exogenous human BMP2. Numbers indicate final concentration of BMP2 in the cell culture medium (in ng/ml). (B) P19 cells were transfected with luciferase reporters containing AmphiVent1, AmphiVent2 and Xvent-2B 5′genomic non-coding regions in the absence of BMP ligand (open bars), or in the presence of either BMP4 (50 ng/ml, grey bars) or BMP7 (50 ng/ml, black bars). (C) P19 cells were transfected with luciferase reporter containing AmphiVent1 5′genomic non-coding region in the absence of ligands (open bar) or presence of human BMP2 (50 ng/ml), human TGF β (20 ng/ml) and human Activin B (10 ng/ml), respectively. **P<0.01, ***P<0.001.
(0.38 MB TIF)

Figure S2 Wnt3A activates Xenopus Xvent-2B but not AmphiVent1 and AmphiVent2 promoters. Indicated luciferase reporter plasmids were transfected into 293T cells in the absence or presence of Wnt3A conditioned medium. Please, note that fold induction of individual reporter genes was normalized to activation of the promoter-less construct pGL3-basic. **P<0.01, ***P<0.001.
(0.10 MB TIF)

Figure S3 Transient expression of EGFP in medaka embryos injected with p817-AmphiVent2. (A-A′) EGFP expression driven by AmphiVent2 promoter at early gastrula stage. (B-C′) EGFP expression patterns in mid-gastrula stage medaka embryos; dorsal (B-B′) and lateral (C-C′) views show EGFP fluorescence in the blastoderm around the most dorsal region of embryonic shield (Sh). Dashed line indicates the borders of the blastoderm. White arrowheads depict the most dorsal embryonic shield of the medaka embryo, where Chordin and Goosecoid are expressed. GR-germ ring, Sh-embryonic shield.
(4.01 MB TIF)

Figure S4 Modulation of TCF/LEF-mediated transcription by Vent proteins. (A) Schematic diagram of TCF/LEF reporter gene pTOPFLASH. (B) TCF/LEF reporter gene pTOPFLASH was cotransfected into 293T cells with CMV-based expression plasmids encoding LEF1, stabilized version of β-catenin (β-cateninΔ) and the indicated Vent protein. Please note that transfection of plasmid encoding AmphiVent1 fusion with strong transcriptional activator VP16 does not lead to detectable activation of pTOPFLASH above vector control. *P<0.05, **P<0.01, ***P<0.001.
(0.09 MB TIF)

Figure S5 Xvent-2B but not AmphiVent1 or AmphiVent2 positively autoregulates its own expression. (A-C) 293T cells were transfected with (A) luciferase reporter containing Xvent-2B promoter in the absence or presence of an expression vector encoding Xvent-2B, (B) luciferase reporters containing AmphiVent1 and AmphiVent2 in the absence or presence of an expression vector encoding AmphiVent1, (C) luciferase reporters containing AmphiVent1 and AmphiVent2 in the absence or presence of an expression vector encoding AmphiVent2. Please note, that only Xvent-2B significantly activates its own promoter. *P<0.05, **P<0.01.
(0.15 MB TIF)
