# Supplementary figures and images for: Conservation and Diversification of an Ancestral Chordate Gene Regulatory Network for Dorsoventral Patterning
Source: PLoS One. 2011 Feb 3;6(2):e14650. doi: 10.1371/journal.pone.0014650 (PMC3033397; doi:10.1371/journal.pone.0014650)

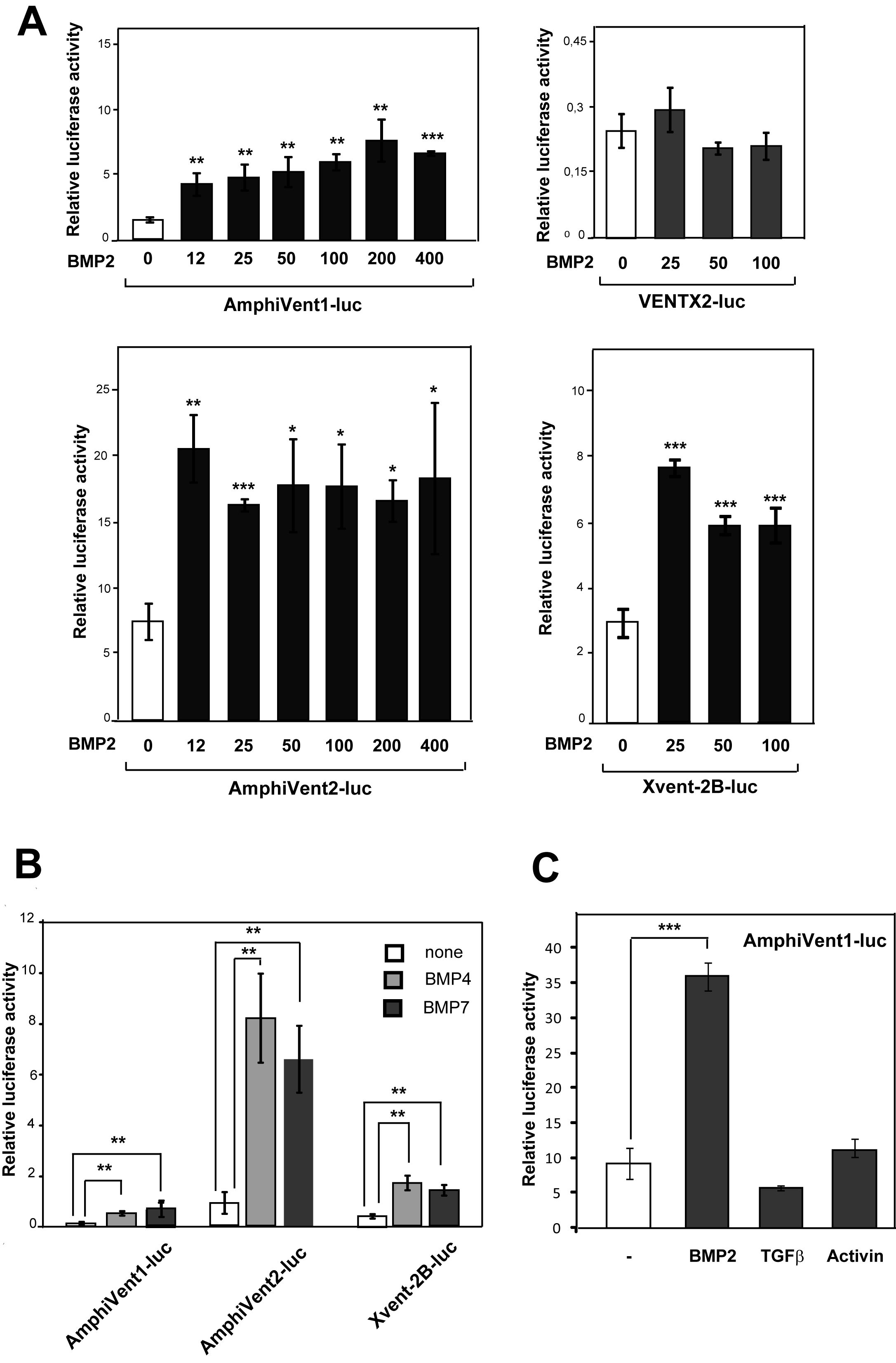

Supplement: Figure S1 — Activation of Amphioxus and Xenopus Vent gene promoters by Bmp2, BMP4 and BMP7. (A) P19 cells were transfected with luciferase reporters containing AmphiVent1, AmphiVent2, Xvent-2B and VENTX2 5′genomic non-coding regions in the absence (open bars) and presence (black bars) of increasing amounts of exogenous human BMP2. Numbers indicate final concentration of BMP2 in the cell culture medium (in ng/ml). (B) P19 cells were transfected with luciferase reporters containing AmphiVent1, AmphiVent2 and Xvent-2B 5′genomic non-coding regions in the absence of BMP ligand (open bars), or in the presence of either BMP4 (50 ng/ml, grey bars) or BMP7 (50 ng/ml, black bars). (C) P19 cells were transfected with luciferase reporter containing AmphiVent1 5′genomic non-coding region in the absence of ligands (open bar) or presence of human BMP2 (50 ng/ml), human TGF β (20 ng/ml) and human Activin B (10 ng/ml), respectively. **P<0.01, ***P<0.001. (0.38 MB TIF) [file pone.0014650.s001.tif]

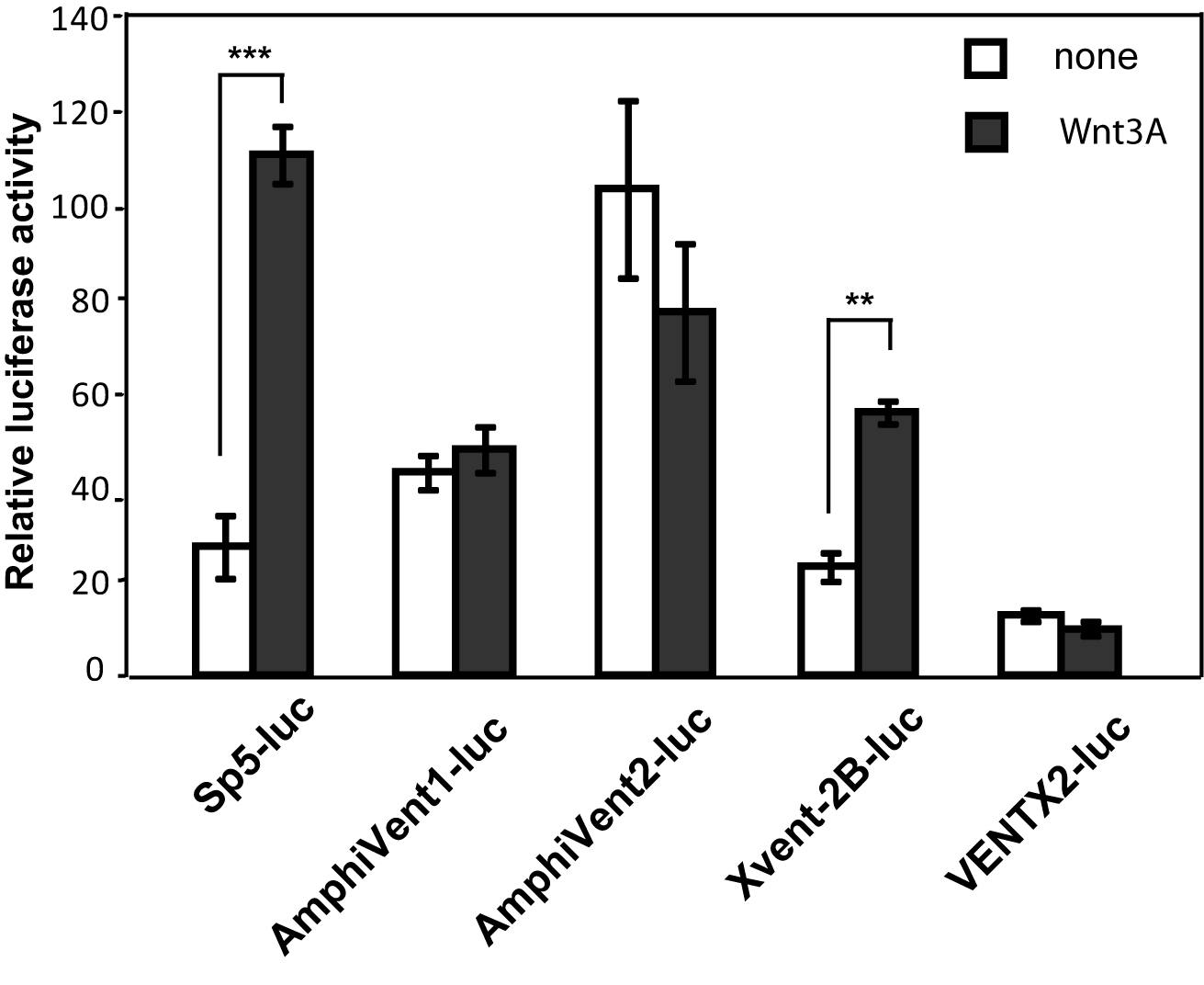

Supplement: Figure S2 — Wnt3A activates Xenopus Xvent-2B but not AmphiVent1 and AmphiVent2 promoters. Indicated luciferase reporter plasmids were transfected into 293T cells in the absence or presence of Wnt3A conditioned medium. Please, note that fold induction of individual reporter genes was normalized to activation of the promoter-less construct pGL3-basic. **P<0.01, ***P<0.001. (0.10 MB TIF) [file pone.0014650.s002.tif]

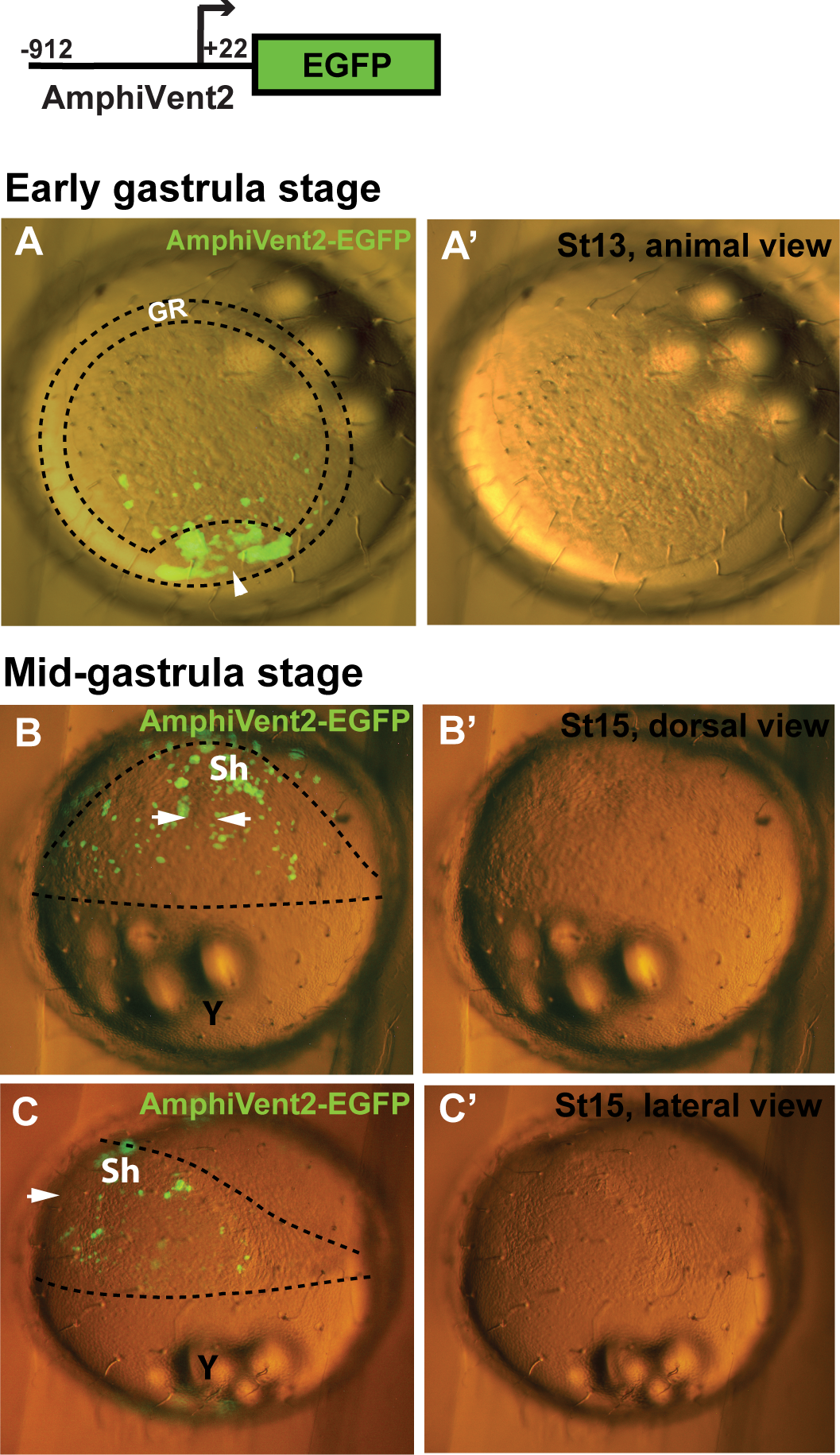

Supplement: Figure S3 — Transient expression of EGFP in medaka embryos injected with p817-AmphiVent2. (A-A′) EGFP expression driven by AmphiVent2 promoter at early gastrula stage. (B-C′) EGFP expression patterns in mid-gastrula stage medaka embryos; dorsal (B-B′) and lateral (C-C′) views show EGFP fluorescence in the blastoderm around the most dorsal region of embryonic shield (Sh). Dashed line indicates the borders of the blastoderm. White arrowheads depict the most dorsal embryonic shield of the medaka embryo, where Chordin and Goosecoid are expressed. GR-germ ring, Sh-embryonic shield. (4.01 MB TIF) [file pone.0014650.s003.tif]

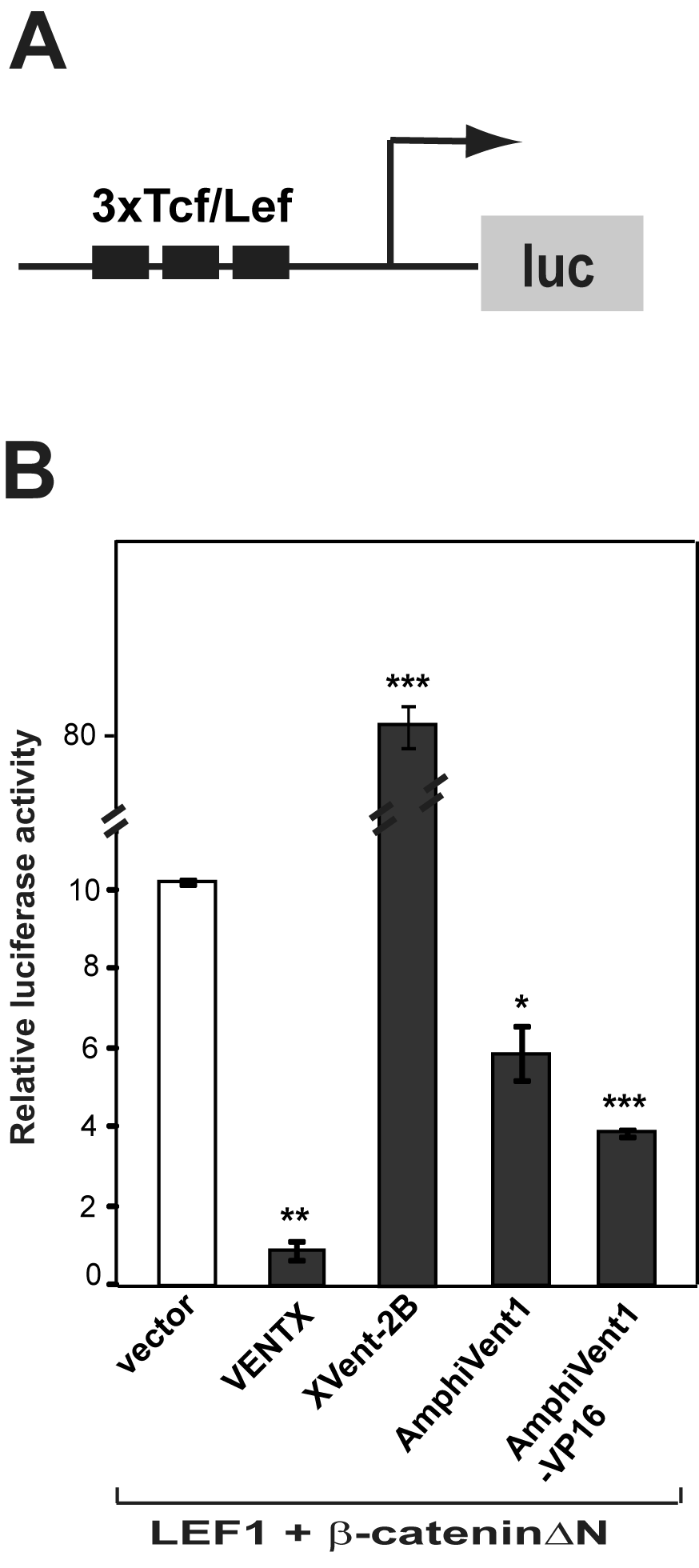

Supplement: Figure S4 — Modulation of TCF/LEF-mediated transcription by Vent proteins. (A) Schematic diagram of TCF/LEF reporter gene pTOPFLASH. (B) TCF/LEF reporter gene pTOPFLASH was cotransfected into 293T cells with CMV-based expression plasmids encoding LEF1, stabilized version of β-catenin (β-cateninΔ) and the indicated Vent protein. Please note that transfection of plasmid encoding AmphiVent1 fusion with strong transcriptional activator VP16 does not lead to detectable activation of pTOPFLASH above vector control. *P<0.05, **P<0.01, ***P<0.001. (0.09 MB TIF) [file pone.0014650.s004.tif]

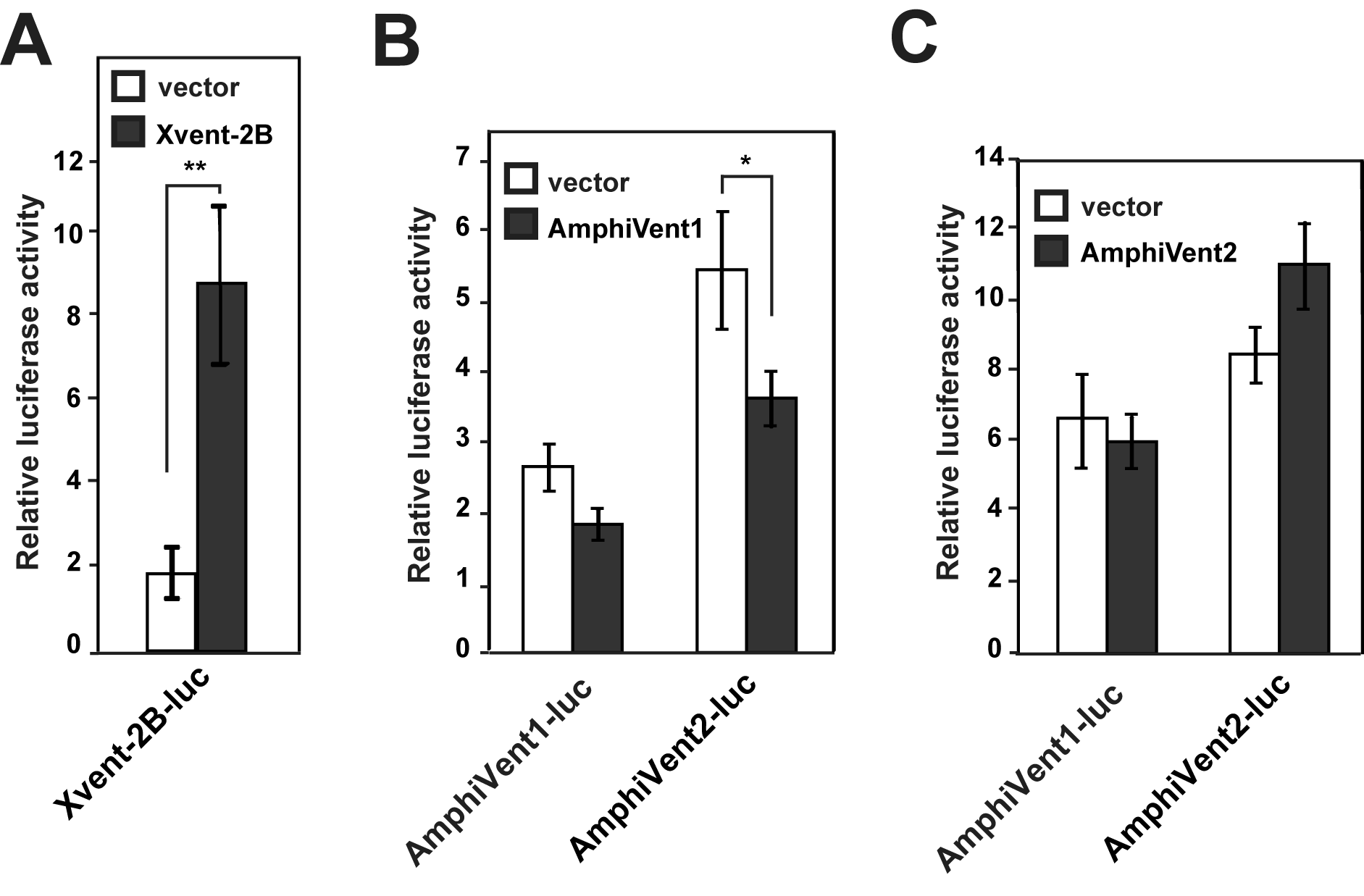

Supplement: Figure S5 — Xvent-2B but not AmphiVent1 or AmphiVent2 positively autoregulates its own expression. (A-C) 293T cells were transfected with (A) luciferase reporter containing Xvent-2B promoter in the absence or presence of an expression vector encoding Xvent-2B, (B) luciferase reporters containing AmphiVent1 and AmphiVent2 in the absence or presence of an expression vector encoding AmphiVent1, (C) luciferase reporters containing AmphiVent1 and AmphiVent2 in the absence or presence of an expression vector encoding AmphiVent2. Please note, that only Xvent-2B significantly activates its own promoter. *P<0.05, **P<0.01. (0.15 MB TIF) [file pone.0014650.s005.tif]
